# Impact of high‐intensity interval training with or without l‐citrulline on physical performance, skeletal muscle, and adipose tissue in obese older adults

**DOI:** 10.1002/jcsm.12955

**Published:** 2022-03-07

**Authors:** Vincent Marcangeli, Layale Youssef, Maude Dulac, Livia P. Carvalho, Guy Hajj‐Boutros, Olivier Reynaud, Bénédicte Guegan, Fanny Buckinx, Pierrette Gaudreau, José A. Morais, Pascale Mauriège, Philippe Noirez, Mylène Aubertin‐Leheudre, Gilles Gouspillou

**Affiliations:** ^1^ Département des sciences biologiques, Faculté des Sciences UQAM Montréal Québec Canada; ^2^ Département des sciences de l'activité physique, Faculté des Sciences UQAM Montréal Québec Canada; ^3^ Groupe de recherche en Activité Physique Adaptée Montréal Québec Canada; ^4^ INSERM U1124, Université de Paris Paris France; ^5^ École de Réadaptation, Faculté de médecine et des sciences de la santé Université de Sherbrooke Sherbrooke Québec Canada; ^6^ Centre de Recherche sur le Vieillissement du Centre intégré universitaire de santé et services sociaux de l'Estrie‐CHUS Sherbrooke Québec Canada; ^7^ Department of Medicine Research Institute of the McGill University Health Centre Montréal Québec Canada; ^8^ Centre de Recherche de l'Institut Universitaire de Gériatrie de Montréal Montréal Québec Canada; ^9^ Département de Médecine de l'Université de Montréal Centre de Recherche du Centre Hospitalier Universitaire de Montréal (CRCHUM), Université de Montréal Montréal Québec Canada; ^10^ Département de kinésiologie Université Laval Québec Québec Canada; ^11^ UFR STAPS Université de Reims Champagne Ardenne Reims France

**Keywords:** High‐intensity interval training, Exercise, Nutrition, Aging, Mobility, Sarcopenia, Obesity, Mitochondrial dynamics, Mitophagy, Mitochondrial quality control, Gene expression

## Abstract

**Background:**

Aging is associated with a progressive decline in skeletal muscle mass and strength as well as an increase in adiposity. These changes may have devastating impact on the quality of life of older adults. Mitochondrial dysfunctions have been implicated in aging‐related and obesity‐related deterioration of muscle function. Impairments in mitochondrial quality control processes (biogenesis, fusion, fission, and mitophagy) may underlie this accumulation of mitochondrial dysfunction. High‐intensity interval training (HIIT) was shown to improve muscle and mitochondrial function in healthy young and old adults and to improve body composition in obese older adults. Recent studies also positioned citrulline (CIT) supplementation as a promising intervention to counter obesity‐related and aging‐related muscle dysfunction. In the present study, our objectives were to assess whether HIIT, alone or with CIT, improves muscle function, functional capacities, adipose tissue gene expression, and mitochondrial quality control processes in obese older adults.

**Methods:**

Eighty‐one‐old and obese participants underwent a 12 week HIIT with or without CIT on an elliptical trainer [HIIT‐CIT: 20 men/25 women, 67.2 ± 5.0 years; HIIT‐placebo (PLA): 18 men/18 women, 68.1 ± 4.1 years]. Handgrip and quadriceps strength, lower limb muscle power, body composition, waist circumference, and functional capacities were assessed pre and post intervention. *Vastus lateralis* muscle biopsies were performed in a subset of participants to quantify markers of mitochondrial content (TOM20 and OXPHOS subunits), biogenesis (TFAM), fusion (MFN1&2, OPA1), fission (DRP1), and mitophagy (Parkin). Subcutaneous abdominal adipose tissue biopsies were also performed to assess the expression of genes involved in lipid metabolism.

**Results:**

HIIT‐PLA and HIIT‐CIT displayed improvements in functional capacities (*P* < 0.05), total (mean ± SD: HIIT‐PLA: +1.27 ± 3.19%, HIIT‐CIT: +1.05 ± 2.91%, *P* < 0.05) and leg lean mass (HIIT‐PLA: +1.62 ± 3.85%, HIIT‐CIT: +1.28 ± 4.82%, *P* < 0.05), waist circumference (HIIT‐PLA: −2.2 ± 2.9 cm, HIIT‐CIT: −2.6 ± 2.5 cm, *P* < 0.05), and muscle power (HIIT‐PLA: +15.81 ± 18.02%, HIIT‐CIT: +14.62 ± 20.02%, *P* < 0.05). Only HIIT‐CIT decreased fat mass (−1.04 ± 2.42%, *P* < 0.05) and increased handgrip and quadriceps strength (+4.28 ± 9.36% and +10.32 ± 14.38%, respectively, *P* < 0.05). Both groups increased markers of muscle mitochondrial content, mitochondrial fusion, and mitophagy (*P* < 0.05). Only HIIT‐CIT decreased the expression of the lipid droplet‐associated protein CIDEA (*P* < 0.001).

**Conclusions:**

High‐intensity interval training is effective in improving functional capacities, lean mass, muscle power, and waist circumference in obese older adults. HIIT also increases markers of mitochondrial biogenesis, mitochondrial fusion, and mitophagy. Importantly, adding CIT to HIIT results in a greater increase in muscle strength and a significant decrease in fat mass. The present study therefore positions HIIT combined with CIT as an effective intervention to improve the health status of obese older adults.

## Introduction

One of the most deleterious hallmarks of normal aging is the progressive loss of muscle mass and strength, a process termed sarcopenia.^1^ Sarcopenia is a major determinant of mobility impairments, falls, physical frailty, and impaired quality of life in older adults.^2^, ^3^, ^4^ Highlighting its serious health consequences, sarcopenia was recognized in 2016 as a muscle condition/disease and received its own ICD (International Classification of Diseases)‐10‐Clinical Modification code (M62.84).^1^ Another major concern for our populations and healthcare systems is the progressive rise in obesity, which is also highly prevalent among older adults.^5^ Importantly, individuals affected by both obesity and sarcopenia display greater decline in physical function vs. individuals affected by obesity or sarcopenia alone.^5^, ^6^, ^7^ Developing strategies to treat or prevent older adults at risk of sarcopenic obesity is therefore one of the major challenges facing health research.

Although the mechanisms underlying the aging‐related loss of muscle mass and function are still partly understood, strong experimental evidence indicates that the accumulation of mitochondrial dysfunction plays an important role in the muscle aging process.^8^ Interestingly, the accumulation of intramuscular lipids secondary to obesity has also been associated with impaired skeletal muscle mitochondrial content and function.^9^ As such, strategies that improve mitochondrial control quality processes might hold promise in fighting sarcopenia^8^, ^10^ and the deleterious consequences of obesity.

Among these strategies, exercise training is well known to improve mitochondrial function and to induce major health benefits.^11^ However, nearly 60% of older adults are sedentary,^12^ with many reporting lack of time as a barrier to do physical activity.^13^ In this context, high‐intensity interval training (HIIT), a time‐efficient subtype of endurance training effective in promoting beneficial adaptations, appears particularly attractive. Indeed, HIIT significantly increases maximal oxygen consumption while reducing cardiometabolic risk factors, including fat mass, in overweight and obese adults.^14^, ^15^, ^16^ HIIT also elicits positive mitochondrial adaptations.^17^, ^18^ Evidence also indicates that short sessions of high‐intensity exercises can induce greater improvements in functional capacities, body composition, and aerobic capacity than moderate‐intensity continuous exercise in older individuals.^15^, ^16^

l‐Citrulline (CIT) supplementation has recently emerged as a potential candidate to improve body composition, muscle function, mitochondrial health, and adipose tissue metabolism in aged rodents and older adults.^19^, ^20^, ^21^, ^22^, ^23^ CIT, a non‐proteinogenic amino acid, is an intermediate of the urea cycle produced in the liver from arginine during nitric oxide production.^19^, ^20^ Importantly, CIT escapes splanchnic extraction.^19^ In aged rodents, CIT supplementation was shown to positively impact muscle mass, fibre size, and the expression and activity of mitochondrial enzymes.^21^ Evidence also indicates that CIT can stimulate fatty acid release from adipocytes, ultimately lowering adipose tissue mass.^22^ In malnourished aged women, CIT supplementation was shown to increase lean mass and decrease fat mass.^23^

Based on the available literature, combining HIIT and CIT supplementation might represent an effective strategy to improve functional capacities, skeletal muscle function, and mitochondrial health, as well as body composition and adipose tissue metabolism, in obese older adults. In support of this view, we recently reported that HIIT combined with CIT induced greater improvements in upper limbs muscle strength and walking speed in obese individual with low muscle strength than HIIT alone.^24^ Similarly, it was reported that whole‐body vibration training combined with CIT induces greater improvement in leg fat‐free mass in obese post‐menopausal women with high blood pressure than whole‐body vibration training alone.^25^

In this setting, the main objectives of the present study were to assess the impact of HIIT with or without CIT supplementation on body composition, functional capacities, muscle strength, and quality in obese older men and women. In a subset of participants that underwent adipose tissues biopsies and muscles biopsies, we have further investigated the impact of HIIT with or without CIT supplementation on the expression of key genes regulating adipose tissue metabolism and muscle protein contents of markers of mitochondrial content, biogenesis, dynamics, and mitophagy.

## Material and methods

### Study design

This is a double‐blind randomized trial (NCT02417428, https://clinicaltrials.gov/ct2/show/NCT02417428). Importantly, the following two groups were removed from the initial registered trial prior to randomization and recruitment: no exercise + placebo (PLA) and no exercise + CIT. The randomization was performed by blocks of four by computer‐generated randomization procedure. All procedures were approved by the Ethics Committee of the Université du Québec à Montréal (UQAM) (#2014_e_1018_475). All participants provided informed written consent after having received information on the nature, goal, procedures, and risks associated with the study.

### Participants

Participants were recruited from the community via social communication (flyers, advertisements in local newspapers, and meetings in community centres) in the Great Montreal area. The detailed list of inclusion and exclusion criteria is available in Supporting Information, Methods S1. A total of 107 participants were recruited. Among them, 95 took part in the study (see *Figure* S1A for a detailed diagram of the study). All participants followed an exercise intervention (HIIT) and received an isocaloric supplementation (CIT or PLA) (*Figure* S1B). Participants were randomly and double blindly assigned to HIIT‐CIT or HIIT‐PLA groups. Eighty‐one participants completed the intervention: HIIT‐PLA (*n* = 36) vs. HIIT‐CIT (*n* = 45) (*Figure* S1A).

### Intervention

#### Exercise training

Participants followed an HIIT on an elliptical trainer (TechnoGym Synchro Exc 700). The latter was chosen to reduce impacts on lower extremity joints.^14^ HIIT was performed three times per week in non‐consecutive days for 12 weeks and was supervised by trained kinesiologists. The intensity of each cycle was based on percentage of maximal heart rate and/or perceived exertion (Borg's scale)^26^ or exclusively based on the latter in case of anti‐arrhythmic and inotropic agents use. The maximal heart rate was determined using the following equation: $\left[\left(\left(220-\mathrm{age}\right)-\text{heart rate rest}\right)\times \%\text{heart rate target}\right]+\text{heart rate rest}$. The 30 min exercise session consisted of a 5 min warm‐up at a low intensity (50–60% maximal heart rate and/or a score between 8 and 12 on Borg's scale); a 20 min HIIT of multiple 30 s sprints at a high intensity (80–85% maximal heart rate or Borg's scale > 17) alternating with 90 s at a moderate intensity (65% maximal heart rate or Borg's scale score 13–16); and a 5 min cool‐down (50–60% maximal heart rate and/or a Borg's scale score 8–12). To ensure that heart rate was always above 80% during high‐intensity intervals, speed and resistance of the elliptical device were continuously adjusted by trained kinesiologists or physiotherapists throughout the training session. Participants needed to complete 80% or more of their training sessions to be included in the per‐protocol analyses.

#### l‐Citrulline supplementation

During the 12 weeks, participants in the HIIT‐CIT group took a single daily dose of 10 g of CIT (Citrage©) containing 38 kcal per dose, while participants in the HIIT‐PLA group took a single dose of a PLA powder (maltodextrin) equivalent in weight, appearance, taste, and calories. Supplements were taken every day during lunch meal. The dose of CIT was based on Moinard *et al*.^21^

### Socio‐demographic and cognitive assessment

Socio‐demographic characteristics (age and sex), cognitive status,^27^ body composition, and aerobic and functional capacities at baseline and at the end of the intervention were assessed for each participant in the same order. The validated Montreal Cognitive Assessment (MoCA) was used to assess cognitive status.^27^ An extra point to the total score is given if the subject has ≤12 years of education.^27^

### Body composition assessment

Body weight and height were determined in fasted state using an electronic scale (Adam GFK 660a) and a stadiometer (Seca), from which body mass index [BMI = body mass (kg)/height (m^2^)] was calculated. Waist circumference was measured to the nearest 0.1 cm. Dual‐energy X‐ray absorptiometry (DXA) (GE Prodigy Lunar) was used to assess fat (total, android, gynoid, and legs) and lean (total, arm, and leg) masses in fasted state.

### Functional and aerobic capacities

The following validated tests were performed: the Timed Up and Go test, the unipodal balance test, the chair stand test, the alternate‐step test, the 6 min walking test, and the 4 m walk test. Detailed procedures for these tests are available in Methods S1.

### Muscle function assessment

Maximum voluntary handgrip strength was measured using a hand dynamometer with adjustable grip (Lafayette Instrument). Maximal quadriceps strength was assessed using a strain gauge system attached to a chair (Primus RS Chair, BTE). Lower limb muscle power was measured using the Nottingham Leg Extensor Power rig with participants in a sitting position. Detailed procedures are provided in Methods S1.

### Energy balance

Detailed protocols used to assess dietary intake and physical activity are provided in Methods S1.

### Blood profiling

Blood samples (15 mL) were collected in the morning after an overnight fast (12 h) to assess fasting serum levels of biochemical and hormonal markers. Venipuncture was performed in participants while seated and blood was collected in gold vacutainer tubes (Becton‐Dickinson, Franklin Lakes, NJ, USA). Details on assays that were used to assess blood profile are available in Methods S1.

### Skeletal muscle biopsies and immunoblotting

A subset of our participants underwent a skeletal muscle biopsy [HIIT‐PLA: *n* = 13 (9 women and 4 men); HIIT‐CIT: *n* = 14 (7 women and 7 men)]. Skeletal muscle samples were obtained from the *vastus lateralis* muscle using the Bergstrom needle biopsy^28^ performed under local anaesthesia. Muscle pieces were snap frozen in liquid nitrogen and stored at −80°C until use.

The protein content of multiple proteins of interest (proteins involved in mitochondrial biogenesis, fusion, fission, and mitophagy; listed in *Table* S1) was determined in muscle homogenates. Detailed immunoblotting procedures are available in Methods S1.

### Abdominal adipose tissue biopsies and quantification of gene expression

A subset of participants underwent an abdominal adipose tissue biopsy [HIIT‐PLA: *n* = 21 (10 women and 11 men); HIIT‐CIT: *n* = 22 (10 women and 12 men)]. Biopsy samples were collected from an area in the lower quadrant (10–12 cm from the umbilicus) using a 12‐gauge Yale needle.^29^

Approximately 1 g of abdominal subcutaneous adipose tissue was collected at the peri‐umbilical region and samples were immediately frozen in liquid nitrogen and kept at −80°C until subsequent analysis of the expression of key genes involved in adipose tissue lipid metabolism. Detailed procedures for gene expression assays are available in Methods S1.

### Statistical analyses

Per‐protocol analyses were conducted. Participants' characteristics at baseline were assessed by unpaired bilateral Student's *t*‐tests or by the Mann–Whitney *U* test when Gaussian distribution was not assumed. Two‐way repeated‐measure ANOVA (if there were no missing values) or mixed‐effects analysis (if there were missing values) were used to assess the effect of time (intervention), group (supplementation), and time * group. Corrections for multiple comparisons following ANOVA or mixed‐effects analysis were performed by Sidak's multiple comparison tests. The percentage change (pre vs. post intervention) was calculated and compared by means of unpaired bilateral Student's *t*‐tests. The impact of our intervention on adipose tissue gene expression was assessed using repeated‐measure mixed‐effects analysis followed by Sidak's multiple comparison tests. All statistical analyses were performed using GraphPad Prism 9.2. Statistical significance was set at the 0.05 probability level.

## Results

### Baseline participants' characteristics

As can be seen in *Table* 1, there was no difference in average age, BMI, waist circumference, fat mass, cognitive status, daily number of steps, and energy intake between HIIT‐PLA and HIIT‐CIT at baseline.

**Table 1:** Participant characteristics at baseline

	HIIT‐PLA	HIIT‐CIT	*P*‐value
Age (years)	68.1 ± 4.1	67.2 ± 4.9	0.353
Men/women (*n*)	20/25	18/18	N/A
BMI (kg/m^2^)	29.3 ± 5.1	29.1 ± 4.3	0.820
Waist circumference (cm)	104.1 ± 12	103.9 ± 11.1	0.957
Total fat mass (%)	37.5 ± 7.9	37.1 ± 6.8	0.782
Android fat mass (%)	46.9 ± 7.5	46.4 ± 7.6	0.756
Gynoid fat mass (%)	39.9 ± 10.1	39.9 ± 9	0.999
Steps per day (*n*)	6457 ± 3094	6254 ± 3034	0.776
Energy intake (kcal/day)	2095 ± 429	1966 ± 317	0.395
MoCA (/30)	27.8 ± 1.5	27.3 ± 1.9	0.290

BMI, body mass index; MoCA, Montreal Cognitive Assessment; N/A, not applicable. Data are mean ± SD.

### The impact of high‐intensity interval training with or without l‐citrulline on functional capacities

**Figure 1:**
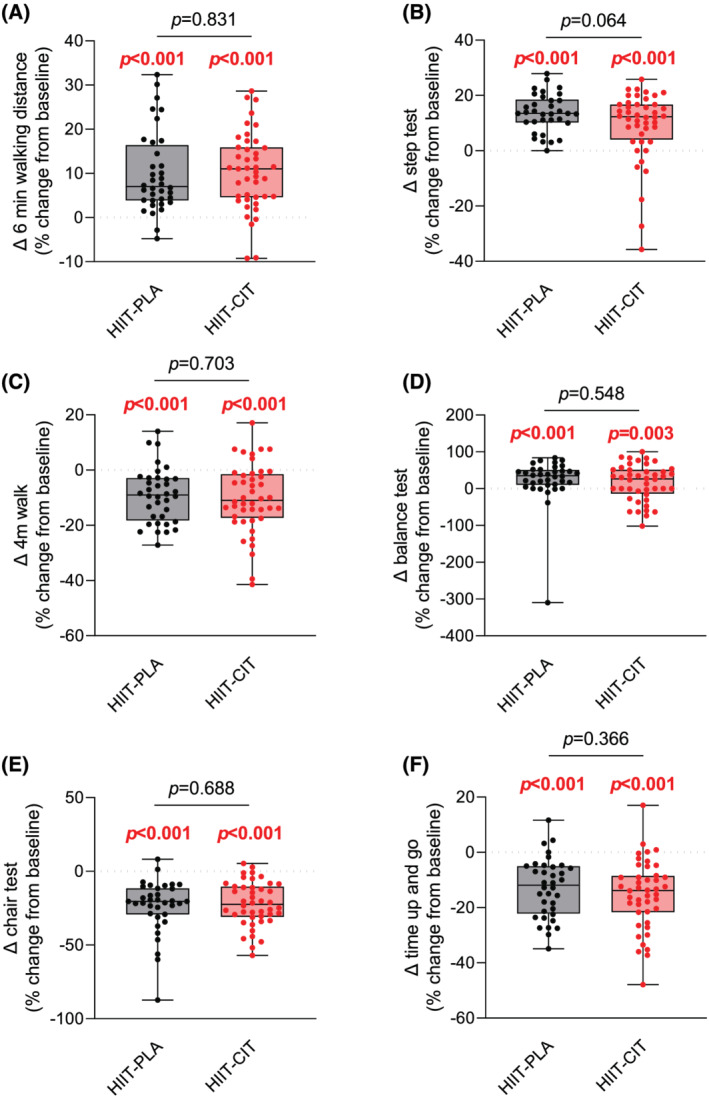
Impact of HIIT with or without CIT on functional capacities. Delta change (in %; pre vs. post) in performance of the HIIT‐CIT and HIIT‐PLA groups at the 6 min walk test *(A)*, step test *(B)*, 4 m walk *(C)*, balance test *(D)*, chair test *(E)*, and Timed Up and Go test *(F)* after 12 weeks of intervention. All significant *P*‐values (*P* < 0.05) are highlighted in bold and in red.

To assess the impact of HIIT with or without CIT on functional capacities, a comprehensive battery of tests was performed. As can be seen in *Table* 2 and *Figure* S2, no difference in functional capacities was observed between HIIT‐PLA and HIIT‐CIT at baseline. Both HIIT‐PLA and HIIT‐CIT displayed significant improvement in their performance at the 6 min walking test, step test, 4 m walk test, balance test, chair test, and Timed Up and Go test (*Figure* 1, *Figure* S2, and *Table* 2). Importantly, no difference in improvement in performance following the proposed 12 week intervention could be evidenced between HIIT‐CIT and HIIT‐PLA (*Figure* 1 and *Table* 2). Similarly, repeated‐measure analyses did not reveal any significant interaction effect for any of the functional capacities' tests between HIIT‐PLA and HIIT‐CIT (*Figure* S2). Taken altogether, these results indicate (i) that HIIT is effective in improving functional capacities in obese older adults and (ii) that CIT does not further improve functional capacities in this population.

**Table 2:** The impact of HIIT with or without CIT on functional capacities, body composition, and skeletal muscle function

	HIIT‐PLA T0	HIIT‐PLA T12	HIIT‐CIT T0	HIIT‐CIT T12	Group effect	Time effect	Time * group effect
**Functional capacities**							
6 min walking test (m)	554 ± 81.6^d^	619 ± 86.3^c^	549 ± 90.6^b^	621 ± 91.2^a^	0.858	**<0.001**	0.809
Step test (*n*)	28.7 ± 3.9^d^	33.2 ± 4.7^c^	29.8 ± 5.2^b^	33.2 ± 6.0^a^	0.657	**<0.001**	0.102
4 m walk test (m/s)	1.9 ± 0.2^d^	2.1 ± 0.3^c^	2.1 ± 0.3^b^	2.1 ± 0.3^a^	0.588	**<0.001**	0.656
Balance test (s)	26.3 ± 18.0^d^	37.1 ± 20.1^c^	27.1 ± 18.7^b^	37.3 ± 21.7^a^	0.991	**<0.001**	0.742
Chair test (s)	19.7 ± 4.9^d^	16.0 ± 3.8^c^	19.5 ± 4.5^b^	16.0 ± 3.9^a^	0.912	**<0.001**	0.571
Timed Up and Go test (s)	7.5 ± 1.0^d^	6.6 ± 0.9^c^	7.5 ± 1.1^b^	6.5 ± 1.3^a^	0.915	**<0.001**	0.861
**Body composition**							
Total lean mass (kg)	47.2 ± 9.6^d^	47.9 ± 10.0^c^	47.3 ± 8.3^b^	47.8 ± 8.6^a^	0.996	**<0.001**	0.717
Legs lean mass (kg)	16.8 ± 3.6^d^	17.1 ± 3.7^c^	16.7 ± 3.0^b^	17.0 ± 3.0^a^	0.896	**0.002**	0.699
Arms lean mass (kg)	5.5 ± 1.8	5.6 ± 1.8	5.3 ± 1.5	5.3 ± 1.5	0.602	0.813	0.839
Total fat mass (%)	37.5 ± 7.9	37.1 ± 7.8^c^	37.1 ± 6.8^b^	36.0 ± 6.5^a^	0.635	**0.003**	0.279
Gynoid fat mass (%)	39.9 ± 10.1	39.6 ± 10.2^c^	39.9 ± 9.0^b^	38.8 ± 9.0^a^	0.862	**0.050**	0.276
Android fat mass (%)	46.9 ± 7.5	46.6 ± 7.7^c^	46.4 ± 7.6^b^	45.0 ± 7.5^a^	0.533	**0.006**	0.109
Waist circumference (cm)	104.1 ± 12.0^d^	101.9 ± 11.6^c^	103.9 ± 11.1^b^	101.5 ± 10.8^a^	0.886	<**0.001**	0.533
**Skeletal muscle function**							
Handgrip strength (kg)	33.0 ± 9.3	34.0 ± 10.3	33.0 ± 10.3^b^	35.0 ± 10.5^a^	0.889	0.001	0.425
Handgrip strength/body weight (kg)	0.41 ± 0.09	0.42 ± 0.1	0.41 ± 0.11^b^	0.44 ± 0.1^a^	0.669	**<0.001**	0.210
Handgrip strength/arms lean mass (kg)	6.2 ± 1.2	2.2 ± 12.3	6.3 ± 1.2^b^	3.4 ± 13.0^a^	0.421	**0.022**	0.418
Quadriceps strength (N)	340.4 ± 92.3	351.4 ± 101.3	322.7 ± 90.5^b^	369.2 ± 94.5^a^	0.970	**<0.001**	**0.023**
Quadriceps strength/body weight (N/kg)	4.2 ± 1.1	4.4 ± 1.0	4.1 ± 1.0^b^	4.7 ± 1.0^a^	0.841	**<0.001**	**0.010**
Quadriceps strength/legs lean mass (N/kg)	20.4 ± 3.6	20.9 ± 3.4	16.6 ± 4.7^b^	21.8 ± 4.1^a^	0.942	**0.002**	0.053
Lower limb power (W)	156.2 ± 71.6^d^	185.8 ± 71.3^c^	154.6 ± 58.3^b^	185.2 ± 64.4^a^	0.907	**<0.001**	0.999

Bold is used to highlight *P*‐values that are below 0.05 (i.e. bold highlights statistically significant differences).

^a^ Significantly different with HIIT‐CIT T0.

^b^ Significantly different with HIIT‐CIT T12.

^c^ Significantly different with HIIT‐PLA T0.

^d^ Significantly different with HIIT‐PLA T12.

### The impact of high‐intensity interval training with or without l‐citrulline on body composition

**Figure 2:**
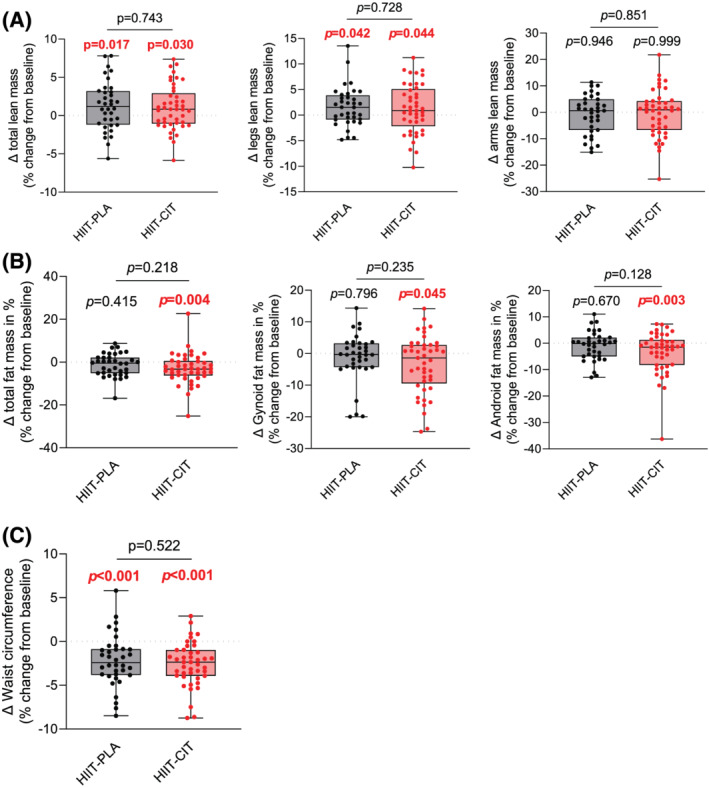
Impact of HIIT with or without CIT on body composition. *(A)* From left to right: delta change (in %; pre vs. post) in total lean mass, legs lean mass, and arms lean mass in HIIT‐CIT and HIIT‐PLA after 12 weeks of intervention. *(B)* From left to right: delta change (in %; pre vs. post) in fat mass, gynoid fat mass, and android fat mass in HIIT‐CIT and HIIT‐PLA after 12 weeks of intervention. *(C)* Delta change (in %; pre vs. post) in waist circumference in HIIT‐CIT and HIIT‐PLA after 12 weeks of intervention. All significant *P*‐values (*P* < 0.05) are highlighted in bold and in red.

To assess the impact of HIIT with or without CIT on body composition, all participants underwent a DXA scan prior to and at the end of the intervention. The waist circumference of all participants was also measured prior to and at the end of the intervention. As can be seen in *Table* 2 and *Figure* S3, there was no difference in total lean mass, leg lean mass, arm lean mass, total fat mass, gynoid fat mass, android fat mass, and waist circumference between HIIT‐CIT and HIIT‐PLA at baseline. Both HIIT‐PLA and HIIT‐CIT displayed increases in total lean mass and leg lean mass at the end of the intervention (*Figure* 2A, *Table* 2, and *Figure* S3A and S3B). Only HIIT‐CIT displayed a decrease in total fat mass, gynoid fat mass, and android fat mass (*Figure* 2B, *Table* 2, and *Figure* S3D–S3F). Both HIIT‐CIT and HIIT‐PLA displayed significant reduction in waist circumference (*Figure* 2C, *Table* 2, and *Figure* S3G). Taken altogether, these data indicate that HIIT is effective in improving lean mass and decreasing waist circumference in obese older adults and that HIIT combined with CIT is an effective intervention to decrease fat mass in this population.

### The impact of high‐intensity interval training with or without l‐citrulline on skeletal muscle strength, power, and quality

**Figure 3:**
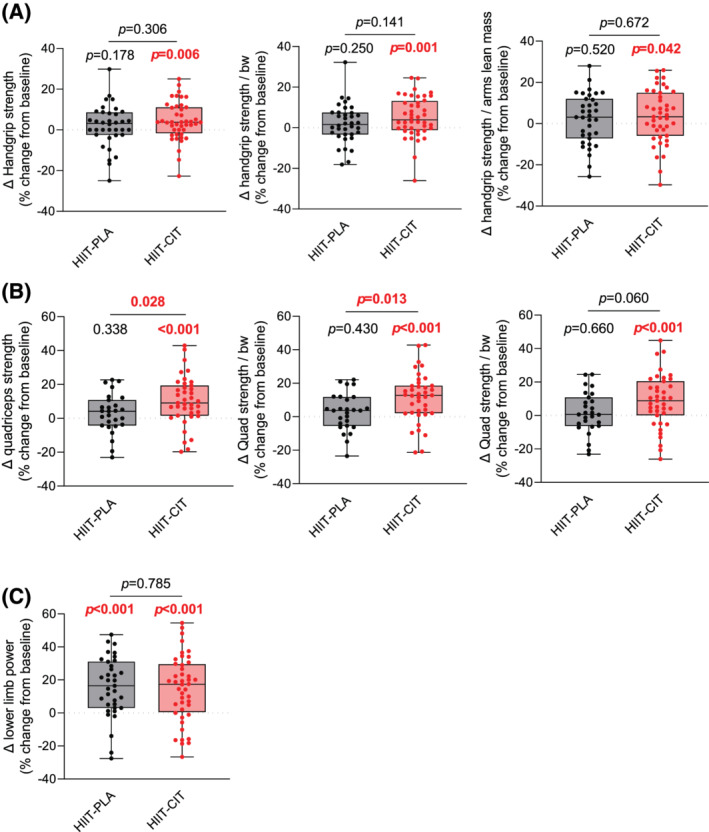
Impact of HIIT with or without CIT on muscle strength, power, and quality. *(A)* From left to right: delta change (in %; pre vs. post) in handgrip strength, handgrip strength normalized to body weight, and handgrip strength normalized to arm lean mass in HIIT‐CIT and HIIT‐PLA after 12 weeks of intervention. *(B)* From left to right: delta change (in %; pre vs. post) in quadriceps strength, quadriceps strength normalized to body weight, and quadriceps strength normalized to legs lean mass in HIIT‐CIT and HIIT‐PLA after 12 weeks of intervention. *(C)* Delta change (in %; pre vs. post) in lower limb power in HIIT‐CIT and HIIT‐PLA after 12 weeks of intervention. All significant *P*‐values (*P* < 0.05) are highlighted in bold and in red.

To assess the impact of HIIT with or without CIT on skeletal muscle strength and power, handgrip strength, lower limb muscle power, and quadriceps strength were evaluated. To assess muscle quality, handgrip strength and quadriceps strength were normalized to body weight and arm or leg lean mass, respectively. As can be seen in *Table* 2 and *Figure* S4, there was no significant difference in handgrip strength, lower limb muscle power, and quadriceps strength between HIIT‐CIT and HIIT‐PLA at baseline. Similarly, no difference in relative handgrip strength (i.e. handgrip strength normalized by body weight or arm lean mass) and in relative quadriceps strength (i.e. quadriceps strength normalized by body weight or leg lean mass) was observed between HIIT‐CIT and HIIT‐PLA at baseline (*Table* 2 and *Figure* S4). While HIIT‐CIT displayed significant increases in absolute and relative handgrip strength, lower limb power, and absolute and relative quadriceps strength, the HIIT‐PLA group only displayed improvement in lower limb muscle power (*Figure* 3, *Table* 2, and *Figure* S4). Repeated‐measure analysis showed an interaction effect for quadriceps strength and quadriceps strength normalized to body weight, indicating that HIIT with CIT is more efficient to increase quadriceps strength than HIIT alone (*Table* 2 and *Figure* S2D and S2E). Taken altogether, these data indicate (i) that HIIT is effective in improving lower limb muscle power in obese older adults and (ii) that HIIT combined with CIT is effective in improving skeletal muscle strength and power in this population.

### The impact of high‐intensity interval training with or without l‐citrulline on serum profile

The impact of HIIT with or without CIT on blood profile is detailed in *Table* S3. At the exception of circulating levels of ferritin that were lower in HIIT‐CIT at baseline—although still in the normal range for men and women—no other difference in blood parameters was observed between HIIT‐PLA and HIIT‐CIT at baseline (*Table* S3). Markers of insulin sensitivity (HOMA‐I and QUICK‐I), circulating levels of IGF1, IGFBP3, total cholesterol, HDL, LDL, free fatty acids, adiponectin, leptin, the adiponectin/leptin ratio, and IGF1/IGFBPP3 molar ratio were unaffected by HIIT‐PLA and HIIT‐CIT (*Table* S3). Surprisingly, HIIT did not improve fasting glycaemia and a small but significant increase in fasting glycaemia was even observed in HIIT‐CIT at the end of the intervention (*Table* S3). Interestingly, circulating levels of triglycerides were decreased at the end of the intervention in the HIIT‐PLA but not in HIIT‐CIT (*Table* S3). Both HIIT‐PLA and HIIT‐CIT lowered circulating levels of ferritin. Taken altogether, the data indicate that HIIT‐PLA and HIIT‐CIT had a very minor impact on blood profile in obese older adults.

### The impact of high‐intensity interval training with or without l‐citrulline on markers of mitochondrial health in skeletal muscles

**Figure 4:**
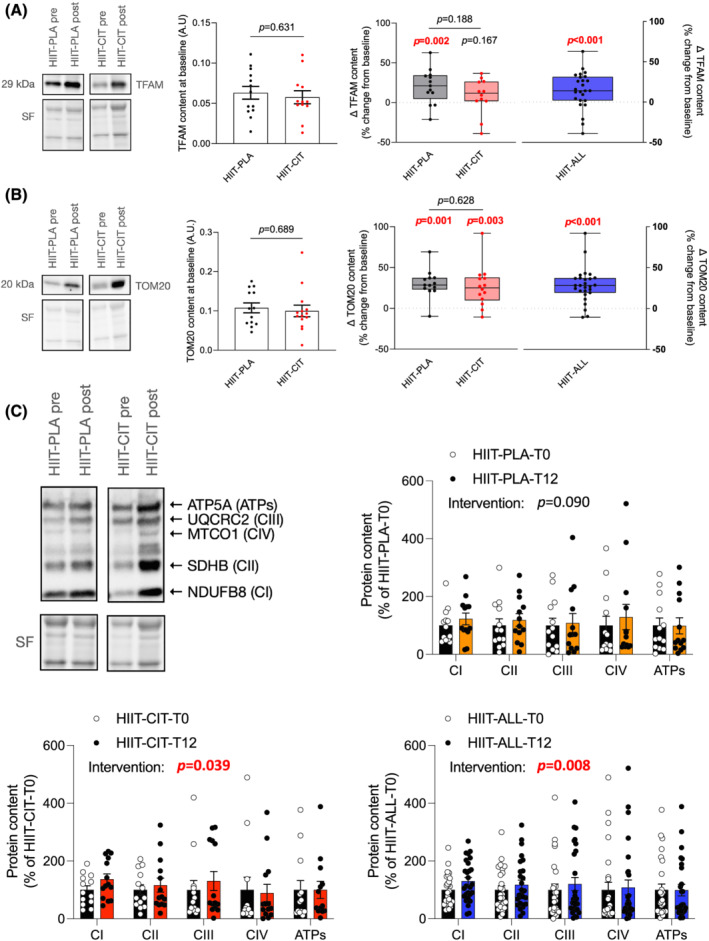
Impact of HIIT with or without CIT on markers of mitochondrial biogenesis and content. *(A)* From left to right: representative TFAM western blot and its corresponding stain free (loading control) for HIIT‐PLA and HIIT‐CIT pre and post intervention; quantification of TFAM content in HIIT‐CIT and HIIT‐PLA at baseline (i.e. pre‐intervention); delta change (in %; pre vs. post) in TFAM content in HIIT‐CIT and HIIT‐PLA after 12 weeks of intervention; delta change (in %; pre vs. post) in TFAM content after 12 weeks of HIIT intervention for all participants (i.e. HIIT‐PLA and HIIT‐CIT combined). *(B)* From left to right: representative TOM20 western blot and its corresponding stain free (loading control) for HIIT‐PLA and HIIT‐CIT pre and post intervention; quantification of TOM20 content in HIIT‐CIT and HIIT‐PLA at baseline (i.e. pre‐intervention); delta change (in %; pre vs. post) in TOM20 content in HIIT‐CIT and HIIT‐PLA after 12 weeks of intervention; delta change (in %; pre vs. post) in TOM20 content after 12 weeks of HIIT intervention for all participants (i.e. HIIT‐PLA and HIIT‐CIT combined). *(C)* Upper left: representative western blot for representative OXPHOS subunits and its corresponding stain free (loading control) for HIIT‐PLA and HIIT‐CIT pre and post intervention; upper right: quantification of OXPHOS subunit contents in HIIT‐PLA pre and post intervention; lower left: quantification of OXPHOS subunit contents in HIIT‐CIT pre and post intervention; lower right: quantification of OXPHOS subunit contents after 12 weeks of HIIT intervention for all participants (i.e. HIIT‐PLA and HIIT‐CIT combined). All significant *P*‐values (*P* < 0.05) are highlighted in bold and in red.

To assess the impact of HIIT with or without CIT on markers of mitochondrial health, a subset of our participants underwent *vastus lateralis* biopsies [HIIT‐PLA: *n* = 13 (9 women and 4 men); HIIT‐CIT: *n* = 14 (7 women and 7 men)]. Muscle samples were used to quantify markers of mitochondrial content (TOM20 and representative subunits of the complexes of the oxidative phosphorylation—OXPHOS) as well as the content of proteins involved in the regulation of mitochondrial biogenesis (TFAM), mitochondrial dynamics (Mfn1, Mfn2, Opa1, and Drp1), and mitophagy (Parkin). No difference in TFAM (*Figure* 4A and *Figure* S5A), TOM20 (*Figure* 4B and *Figure* S5B), and OXPHOS subunit (*Figure* 4C) contents was observed between HIIT‐PLA and HIIT‐CIT at baseline. HIIT‐PLA displayed a significant increase in TFAM content at the end of the intervention (*Figure* 4A and *Figure* S5A). Both HIIT‐PLA and HIIT‐CIT displayed a significant increase in TOM20 after 12 weeks of intervention (*Figure* 4B and *Figure* S5B). Only HIIT‐CIT displayed a significant increase in OXPHOS subunit content at the end of the intervention (*Figure* 4C), although a trend for an increase was also observed in HIIT‐PLA (*P* = 0.08994; *Figure* 4C). No difference in the delta change (change from pre‐intervention to post‐intervention) in TFAM, TOM20, and OXPHOS subunit contents was observed between HIIT‐PLA and HIIT‐CIT. Acknowledging the possibility of a type‐II error due to our limited sample size, data from HIIT‐PLA and HIIT‐CIT were combined to assess the impact of exercise alone (HIIT‐ALL) on these markers. As can be seen in *Figure* 4 and *Figure* S5, combining data from HIIT‐CIT and HIIT‐PLA indicates that HIIT is an effective intervention in increasing markers of mitochondrial biogenesis (TFAM) and mitochondrial content (TOM20 and OXPHOS subunits) in obese older adults.

**Figure 5:**
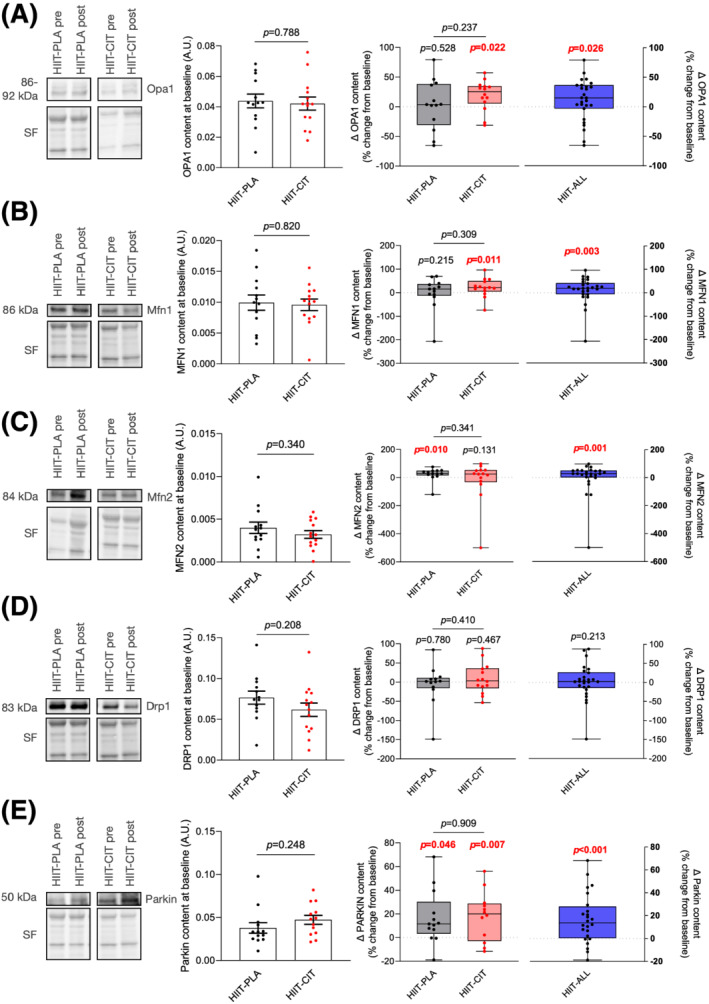
Impact of HIIT with or without CIT on markers of mitochondrial fusion and fission. *(A)* From left to right: representative Opa1 western blot and its corresponding stain free (loading control) for HIIT‐PLA and HIIT‐CIT pre and post intervention; quantification of Opa1 content in HIIT‐CIT and HIIT‐PLA at baseline (i.e. pre‐intervention); delta change (in %; pre vs. post) in Opa1 content in HIIT‐CIT and HIIT‐PLA after 12 weeks of intervention; delta change (in %; pre vs. post) in Opa1 content after 12 weeks of HIIT intervention for all participants (i.e. HIIT‐PLA and HIIT‐CIT combined). *(B)* From left to right: representative Mfn1 western blot and its corresponding stain free (loading control) for HIIT‐PLA and HIIT‐CIT pre and post intervention; quantification of Mfn1 content in HIIT‐CIT and HIIT‐PLA at baseline (i.e. pre‐intervention); delta change (in %; pre vs. post) in Mfn1 content in HIIT‐CIT and HIIT‐PLA after 12 weeks of intervention; delta change (in %; pre vs. post) in Mfn1 content after 12 weeks of HIIT intervention for all participants (i.e. HIIT‐PLA and HIIT‐CIT combined). *(C)* From left to right: representative Mfn2 western blot and its corresponding stain free (loading control) for HIIT‐PLA and HIIT‐CIT pre and post intervention; quantification of Mfn2 content in HIIT‐CIT and HIIT‐PLA at baseline (i.e. pre‐intervention); delta change (in %; pre vs. post) in Mfn2 content in HIIT‐CIT and HIIT‐PLA after 12 weeks of intervention; delta change (in %; pre vs. post) in Mfn2 content after 12 weeks of HIIT intervention for all participants (i.e. HIIT‐PLA and HIIT‐CIT combined). *(D)* From left to right: representative Drp1 western blot and its corresponding stain free (loading control) for HIIT‐PLA and HIIT‐CIT pre and post intervention; quantification of Drp1 content in HIIT‐CIT and HIIT‐PLA at baseline (i.e. pre‐intervention); delta change (in %; pre vs. post) in Drp1 content in HIIT‐CIT and HIIT‐PLA after 12 weeks of intervention; delta change (in %; pre vs. post) in Drp1 content after 12 weeks of HIIT intervention for all participants (i.e. HIIT‐PLA and HIIT‐CIT combined). *(E)* From left to right: representative Parkin western blot and its corresponding stain free (loading control) for HIIT‐PLA and HIIT‐CIT pre and post intervention; quantification of Parkin content in HIIT‐CIT and HIIT‐PLA at baseline (i.e. pre‐intervention); delta change (in %; pre vs. post) in Parkin content in HIIT‐CIT and HIIT‐PLA after 12 weeks of intervention; delta change (in %; pre vs. post) in Parkin content after 12 weeks of HIIT intervention for all participants (i.e. HIIT‐PLA and HIIT‐CIT combined). All significant *P*‐values (*P* < 0.05) are highlighted in bold and in red.

As can be seen in *Figure* 5 and *Figure* S7, the content of proteins regulating mitochondrial dynamics (Mfn1, Mfn2, Opa1, and Drp1) and mitophagy (Parkin) was comparable between HIIT‐PLA and HIIT‐CIT at baseline. HIIT‐CIT displayed a significant increase in the pro‐fusion proteins OPA1 and Mfn1 (*Figure* 5A and 5B and *Figure* S7A and S7B) while HIIT‐PLA displayed a significant increase in the pro‐fusion Mfn2 protein (*Figure* 5C and *Figure* S7C). Neither HIIT‐CIT nor HIIT‐PLA altered the content of the pro‐fission Drp1 protein (*Figure* 5D and *Figure* S7D). Both HIIT‐PLA and HIIT‐CIT increased Parkin content (*Figure* 5E and *Figure* S7E). Acknowledging the possibility of a type‐II error due to our limited sample size, data from HIIT‐PLA and HIIT‐CIT were combined to assess the impact of exercise alone (HIIT‐ALL). As can be seen in *Figure* 5 and *Figure* S7, HIIT is an effective intervention in increasing markers of mitochondrial fusion (OPA1, Mfn1, and Mfn2) and mitophagy (Parkin) but does not impact the content of the mitochondrial fission protein Drp1 in obese older adults.

### The impact of high‐intensity interval training with or without l‐citrulline on gene expression in abdominal subcutaneous adipose tissue

**Figure 6:**
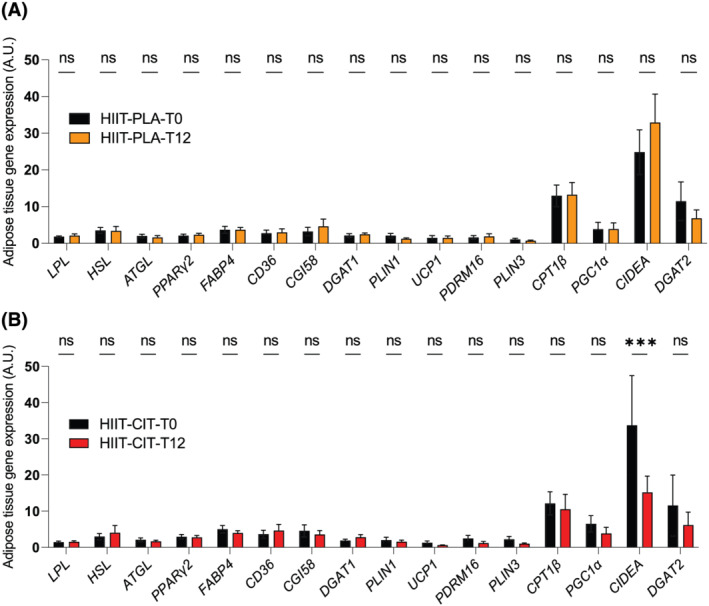
Impact of HIIT with or without CIT on adipose tissue gene expression. Adipose tissue gene expression pre and post intervention in HIIT‐PLA *(A)* and HIIT‐CIT *(B)*. ns, non‐significant; ***, *P* < 0.001.

Because HIIT was previously reported effective in lowering fat mass^30^ and because preclinical data have indicated that CIT can trigger lipolysis in adipocytes,^22^ biopsies were performed in the abdominal subcutaneous adipose tissue to assess the expression of key genes involved in adipose tissue metabolism in a subset of our participants [HIIT‐PLA: *n* = 21 (10 women and 11 men); HIIT‐CIT: *n* = 22 (10 women and 12 men)]. The samples were then used to quantify the expression of various essential genes involved in adipocyte metabolism: lipolysis (*HSL*, *ATGL*), lipid intake (*LPL*), adipocytes differentiation (*PPARγ2*), lipid transport (*FABP4*, *CD36*), lipid droplet homeostasis (*PLIN1*, *PLIN3*, *CGI58*, *CIDEA*), adipocyte browning (*UCP1*, *PDRM16*), lipid oxidation (*CPT1B*), transcriptional regulation of adipocyte metabolism (*PGC‐1α*), and lipogenesis (*DGAT2*) (*Figure* 6). Surprisingly, none of the genes we probed for displayed altered expression in the HIIT‐PLA group at the end of the intervention (*Figure* 6A). Only CIDEA, a gene coding for a lipid droplet‐associated protein,^31^ was found down‐regulated in the HIIT‐CIT group at the end of the intervention (*Figure* 6B). Taken altogether, these data indicate that 12 weeks of HIIT with or without CIT had limited impact on adipose tissue gene expression.

## Discussion

Considering the high prevalence of sarcopenia and obesity in older adults, designing effective strategies to improve muscle mass and function and lower adiposity is urgent. In this setting, our study appears particularly important as it provides evidence (i) that HIIT is effective in improving functional capacities, lean mass, muscle power, and waist circumference in obese older adults and (ii) that HIIT increases markers of mitochondrial biogenesis, mitochondrial fusion, and mitophagy in this population. Importantly, our data also indicate that adding CIT to HIIT confers some additional benefits because HIIT combined with CIT results in greater increases in muscle strength and muscle quality and a significant decrease in fat mass.

The present study extends to the growing body of literature showing that HIIT is an efficient strategy to improve the health status of older adults and obese individuals. Consistent with our findings, it was indeed reported that HIIT can improve skeletal muscle power^32^ and fat‐free mass in older adults.^18^ Similarly, the significant reduction in waist circumference reported herein following HIIT—a parameter closely associated with cardiometabolic risk^33^—and the decrease in circulating levels of triglycerides are in line with recent meta‐analyses showing that HIIT is an efficient intervention to improve cardiometabolic health in obese individuals.^34^, ^35^ Importantly, the significant increase in muscle strength and decrease in fat mass reported in this study in the HIIT‐CIT indicate that CIT supplementation confers additional benefit to HIIT in obese older adult. Consistent with these findings, our group recently reported that HIIT combined with CIT induced greater improvements in upper limbs muscle strength and walking speed in obese individuals with low muscle strength when compared with HIIT alone.^24^ The present study therefore positions HIIT combined with CIT as an effective, safe, and well‐tolerated intervention to improve functional capacities, muscle strength and power, and lower fat mass in obese older adults.

Based on previous reports that have shown that HIIT can lower fat mass^30^ and that CIT can trigger lipolysis in old rats' adipocytes,^22^ we expected that HIIT alone and HIIT combined with CIT would impact the expression of key genes involved in adipose tissue metabolism. Surprisingly, we found that HIIT alone did not lead to a significant decrease in fat mass and had no impact on the expression of any of the gene we probed for. While HIIT combined with CIT leads to a significant reduction in fat mass, this combined intervention only lowered the expression of CIDEA. This result is particularly intriguing because CIDEA is positively associated with metabolic health.^36^ Nonetheless, and to our surprise, our results suggest that the transcriptional impact of HIIT alone or combined with CIT on adipose tissue gene expression is limited considering the set of genes studied. They also suggest that the positive impact of CIT on fat mass is not associated with a major transcriptional reprogramming in adipocytes. However, these data should be considered with caution as multi‐omics approaches would be required to conclude on the impact of HIIT alone or combined with CIT on metabolic adaptations in human adipocytes.

Previous studies in both animal and humans have reported that CIT can stimulate protein synthesis, potentially through an activation of the mTOR pathway (see literature^19^ for a detailed review). Because muscle mass is regulated by the balance between protein synthesis and degradation, one could have expected CIT to positively impact muscle mass. In line with this view, it was recently reported that whole‐body vibration training combined with CIT induced greater improvement in leg fat‐free mass in obese post‐menopausal women than whole‐body vibration training alone.^25^ In malnourished older women, CIT supplementation was also shown to increase lean mass and decrease fat mass.^23^ However, our data contrast with these previous reports as no additional effect of CIT on whole body lean mass, leg lean mass, or arm lean mass could be evidenced in the present study. While differences in studied population (nutritional status, inclusion of both men and women in the present study, etc.) likely contributed to these divergent findings, it is also possible that any effect of CIT might have been masked by the positive impact of HIIT on whole body lean mass and leg lean mass.

In the last few decades, accumulation of mitochondrial dysfunction has emerged as a key mechanism contributing to the muscle aging process^8^ and accumulation of intramuscular lipids secondary to obesity has been associated with impaired skeletal muscle mitochondrial content and function.^9^ In this setting, HIIT appears as a promising therapeutic approach as studies conducted in young healthy individuals clearly demonstrated that HIIT is an effective intervention to increase mitochondrial content and function in skeletal muscles.^37^, ^38^, ^39^ However, studies on the impact of HIIT on mitochondrial content in obese older adults remain scarce. The data presented herein provide evidence that HIIT effectively increases mitochondrial content in the muscle studied in this population. Our data are in line with recent studies that have shown that HIIT is effective in increasing mitochondrial content and respiration in older adults.^18^, ^40^ Importantly, our results also indicate that HIIT positively impacts processes in charge of mitochondrial quality control in obese older adults. Indeed, HIIT increased markers of mitochondrial biogenesis, mitochondrial fusion, and mitophagy. No significant additional effect of CIT could be evidenced in the present study on markers of mitochondrial biogenesis, mitochondrial dynamics, or mitophagy (i.e. absence of difference in per cent change between HIIT‐PLA and HIIT‐CIT and absence of interaction effect). Taken altogether, these results therefore indicate that HIIT is an effective intervention to optimize mitochondrial health in obese older adults.

To our knowledge, the present study is the first to provide a thorough *in vivo* assessment of the impact of HIIT with or without CIT on physical performance, body composition, and muscle function coupled with a detailed exploration of the cellular and molecular adaptations triggered by these interventions in skeletal muscle and adipose tissue of obese older men and women. However, some limitations should be noted. First, the absence of a control group receiving only CIT (without HIIT) prevented the identification of the specific impact of CIT on muscle and adipose cells. Whether CIT alone can confer benefits to obese older adults therefore remains unknown. Second, our limited sample size prevented the exploration of whether there was a sex specificity in the cellular and molecular adaptations to HIIT with or without CIT. Third, our exploration of the impact of HIIT with or without CIT on adipose tissue gene expression was limited to a narrow number of genes regulating lipid metabolism. Future studies should consider using unbiased and high‐throughput approaches, such as RNA‐sequencing, to provide a complete picture of the impact of HIIT with or without CIT on the transcriptome of adipose cells.

## Conclusions

The present study provides evidence (i) that HIIT is safe and well tolerated in obese older adults, (ii) that HIIT is effective in improving functional capacities, lean mass, muscle power, and waist circumference in obese older adults, and (iii) that HIIT increases markers of mitochondrial biogenesis, mitochondrial fusion, and mitophagy in this population. Importantly, our data also indicate that adding CIT to HIIT results in a greater increase in muscle strength and a significant decrease in fat mass. The present study therefore positions HIIT combined with CIT as an effective intervention to improve indicators of cardiovascular and metabolic health of obese older adults.

## Conflict of interest

No potential conflict of interest was reported by the authors.

## Funding

This work was funded by grants from the Quebec Research Network on Aging of the Fonds de Recherche en Santé du Québec (FRQS) awarded to M.A.L., G.G., P.G., J.M., and P.N. and a Canadian Institutes of Health Research (CIHR) grant awarded to G.G., M.A.L., P.G., and J.M. G.G. is supported by a Chercheur Boursier Junior 2 salary award from the FRQS. M.A.L. is supported by a Chercheur Boursier Senior salary award from the FRQS.

## Supporting information


**Data S1.** Supporting information

**Figure S1:** Study Overview
**Figure S2:** Impact of HIIT with or without CIT on functional capacities
**Figure S3:** Impact of HIIT with or without CIT on body composition
**Figure S4:** Impact of HIIT with or without CIT on muscle strength and power and quality
**Figure S5:** Impact of HIIT with or without CIT on TFAM and TOM20 content
**Figure S6:** OXPHOS subunit content in HIIT‐PLA and HIIT‐CIT at baseline (pre‐intervention)
**Figure S7:** Impact of HIIT with or without CIT on markers of mitochondrial dynamics and mitophagy

**Table S1:** List of antibodies
**Table S2:** Primer sequences and qPCR conditions for genes of interest.
**Table S3:** Impact of HIIT with and without CIT on blood parameters
