# Supplementary material for: Impact of high‐intensity interval training with or without l‐citrulline on physical performance, skeletal muscle, and adipose tissue in obese older adults
Source: J Cachexia Sarcopenia Muscle. 2022 Mar 7;13(3):1526–40. doi: 10.1002/jcsm.12955 (PMC9178162; doi:10.1002/jcsm.12955)
Supplement: Supplementary file 1 — Data S1. Supporting information [file JCSM-13-1526-s003.docx]

*Supplemental 1: Inclusion and exclusion criteria:*

To be included in this study, subjects had to meet the following criteria: 1- autonomous (being able to follow the exercise program), 2- obesity (BMI between 30 and 40 kg / m2) or a waist circumference greater than 102 cm for men and 88 cm for women or fat mass (%; total or androïd or gynoïd) equal or superior to 27% in men and 40% in women; 3- stable weight (± 5 kg) for 6 months; 4- non-smokers and moderate drinkers (max: 15 g/day of alcohol) ; 5- no history of stroke ; 6- inactive (less than 2 hours of structured physical activity per week); 7- no involvement in a vigorous exercise program for at least 12 months; 8- ability to understand French; 9- postmenopausal for women (over 60 consecutive months without menses). Exclusion criteria were the following: 1- presence of metal implant (pacemaker); 2- asthma requiring oral steroid treatment; 3-use of medication that could affect metabolism or cardiovascular function; 4- use of anticoagulants (only for participants undergoing biopsies); 5- use of hormonal-replacement therapy. Participants with diagnosed (untreated) neurological, cardiovascular, lung diseases or cognitive disorders were excluded.

*Functional and Aerobic Capacities*

Six different validated tests were performed to assess functional capacities:

- Walking speed was estimated using the “Timed Up & Go” test (in seconds). This validated test [1], which consists in standing from a chair, walking a 3 m distance and sitting down again [2], was performed at a comfortable and self-paced and at a fast-paced walking speed. A duration above 30 s indicates limited mobility and an increased risk of falling whereas a duration of less than 20 s indicates appropriate mobility with subject likely to be independent in activities of daily living [3].
- Balance was assessed using the unipodal balance test. This test has extensive clinical support with a good test-retest reliability [4]. Participants were standing on both legs and alternately standing on the right and left leg with eyes opened and arms by the side of the trunk. The time was recorded in seconds from the moment one foot was lifted from the floor to the moment when it touched the ground, the stance leg moved, or until 60 s had elapsed [5].
- Lower-body function was measured using the chair stand test. Participants were asked to stand up from a sitting position and to sit down 10 times as fast as possible, with arms across their chests [6]. This test is reproducible and correlates with lower extremity muscle strength [7]. The time (in seconds) to perform the task was recorded.
- Weight shifting ability in the forward and upward directions was estimated using the reliable and validated alternate-step test [8, 9]. Participants were placed facing toward a 20 cm height step and instructed to touch its top with the right and left foot, alternatively, as fast as possible during a 20 s period [8, 9]. The number of steps was recorded for analysis.
- Mobility and aerobic capacities were evaluate using the validated 6 min walking test [10, 11]. Participants were asked to walk as much as possible during 6 min. In each minute of the test, volunteers received the same standardized encouragement according to the ATS/American College of Chest Physicians recommendations for the six-minute walking test [11, 12]. Participants were allowed to interrupt and return to exercising as well as to reduce or increase speed according to perceived effort [11].
- To assess walking speed, participants were asked to walk 4 m as fast as possible. This test has been validated [13, 14]. Data were expressed in m.s^-1^.

*Muscle Function Assessment*

Maximum voluntary handgrip strength was measured using a hand dynamometer with adjustable grip (Lafayette Instrument), as previously described [15]. Participants were standing upright with the arm along the side of the body with the elbow extended and the palm of the hand facing the thigh. Participants were advised to squeeze as hard as possible the hand dynamometer for up to 4 s. Three measurements for each hand, alternatively, were performed and the maximal score for each was recorded. Handgrip strength was expressed in absolute (kg) and relative to body weight (BW; kg/kg) and relative to arm lean mass (kg/kg).

Maximal quadriceps strength was assessed using a strain gauge system attached to a chair (Primus RS Chair, BTE) upon which participants were seated with the knee and hip joint angles set at 135◦ and 90◦, respectively. The knee angle was set to 135◦, compared to the typical 90◦, in order to diminish the maximal joint torque that could be generated, particularly in light of generally more fragile bones in older adults. The tested leg was fixed to the lever arm at the level of the lateral malleoli on an analog strain gauge to measure strength. The highest of three maximum voluntary contractions was recorded. Quadriceps strength was expressed in absolute terms (N), relative to body weight (N/kg) and relative to leg lean mass (N/kg).

Lower limb muscle power was measured using the Nottingham Leg Extensor Power rig with participants in a sitting position. Participants were asked to push the pedal down as hard and fast as possible, accelerating a flywheel attached to an analog to digital converter. Power was recorded for each push until a plateau/decrease was observed. This validated assessment has been demonstrated to be safe, sensitive, and reliable in older adults [16, 17].

*Energy Balance*

As previously described and validated in older adults, dietary intake was assessed before and after the intervention using the 3-day food record method (two weekdays and one weekend day) [18]. Participants were asked to keep their dietary habits during the intervention period. Analyzes of total energy intake were performed using the software Nutrific©.

The number of steps were used to estimate the level of physical activity using a validated tri-axial accelerometer (SenseWear®Mini Armband) as previously described [19]. Participants had to wear the device on the left arm all the time during 3 consecutive days, except when taking a shower or swimming. Each participant had to wear the device at least 85% of the time to be included in the study.

*Blood profiling*

Free Fatty Acids (FFA) and glucose were measured using colorimetric enzymatic assays (detection limits 0.005 and 0.01mmol.L^-1^, respectively; Fugifilm Wako DiagnosticUSA Corp., Mountain View, CA, US). Insulin was measured using a human insulin enzyme-linked immunosorbent (ELISA) kit (Mercodia ELISA assay, Uppsala, Sweden; detection limit: 6pmol.L^-1^ per tube; intra- assay coefficient of variation: 3.7%). Homeostatic model assessment insulin resistance index (HOMA-IR) was calculated using the formula: fasting serum insulin (pmol.L^-1^) x 0.167 x glucose (mmol.L^-1^)/22.5. Cholesterol profile (total cholesterol, HDL-C and LDL-C) was evaluated at the McGill University Health Center. Levels of total IGF-1 (intra-assay CV: 5.9 ± 0.6%; detection limit: 0.03 ng/mL) and IGFBP3 (intra-assay CV: 3.9 ± 0.4%; detection limit: 0.14 ng/mL) were measured in a single assay using human ELISA kits (IGF-1 DG100; IGFBP3 DGB300) from R&D Systems Inc. (Minneapolis, MN, USA). The IGF-1/IGFBP-3 molar ratio was calculated considering that 1 ng.mL^-1^ IGF-1 = 0.130 nmol IGF-1 and 1 ng.mL^-1^ IGFBP3 = 0.036 nmol IGFBP-3. Serum leptin (intra-assay CV: 5.5 ± 0.5%; detection limit: 0.5 ng.mL^-1^) and total adiponectin (intra-assay CV: 3.9 ± 0.4%; detection limit: 0.78 ng/mL) were also measured in a single assay using human ELISA kits (leptin EZHL-80SK; adiponectin EZHADP-61K) from EDS Millipore (Burlington, MASS, USA).

*Immunoblotting*

Approximately 15-30 mg of muscle tissue were homogenized in 10 volumes of an extraction buffer composed of tris base 50mM, NaCl 150mM, triton X-100 1%, sodium deoxycolate 0.5%, SDS 0.1% and 10μl/ml of a protease inhibitor cocktail (Sigma P8340), as previously described [20-22]. The homogenate was centrifuged at 15,000g for 15 minutes at 4°C. Protein content in the supernatant was determined using the Bradford method. Aliquots of supernatant were mixed with Laemli buffer and subsequently boiled at 95°C for 5 minutes. Approximately 20μg of proteins were loaded onto gradient (4-15%) and stain-free gels (Mini PROTEAN® TGX Stain-Free TM Gels, Biorad), electrophoresed by SDS-PAGE and then transferred to polyvinylidene fluoride membranes (PVDF, Biorad). A stain-free blot image was taken using the ChemiDocTM Touch Imaging System for total protein measurement in each sample lane. Membranes were blocked in PBS + 1% Tween® 20 + 5% BSA for 1 hour at room temperature and then incubated with the specific primary antibodies for 1 hour. The complete list of antibodies used for immunoblotting analyses can be found in Table 1. All antibodies were diluted in blocking buffer. Membranes were washed in TBS-T (3x5 minutes) and incubated with HRP-conjugated secondary anti-rabbit or anti-mouse secondary antibodies (Abcam, cat# Ab6728, Ab6721) for 1 hour at room temperature, before further washing in TBS-T (3x5 minutes). Signals were detected using enhanced chemiluminescence substrate (Biorad, Clarity ECL substrate, 170-5060) using the ChemiDocTM Touch Imaging System. All images were analyzed using the ImageLab software (Biorad). For each sample, the ECL signal for the protein of interest was normalized to the intensity of the stain-free blot image of the corresponding sample (i.e., the intensity of the stain-free blot image was used as a loading control).

*Quantification of gene expression*

Briefly, about 5 to 10µg of total RNA was extracted from 100 mg of AT, using RNeasy Mini Kit (Qiagen, Mississauga, ON, Canada) according to the manufacturer’s standard procedure. This technique integrates phenol-guanidine lysis and silica gel-membrane purification of total RNA. The isolated total RNA concentration was quantified by absorbance at 260 nm, and the 260/280 nm absorption ratio of all preparations ranged between 1.9 and 2.1. One µg of total RNA was reverse transcribed into complementary DNA (cDNA) using Super Script II Reverse Transcriptase kit (Invitrogen Canada, Burlington, ON), and 50 ng of cDNA were used for real-time quantitative polymerase chain reaction (RT-qPCR). AT mRNA levels were quantified by fluorescent quantitative PCR on a Rotor Gene 3000 (Corbett Research, Montréal Biotech, Kirkland, QC, Canada), using the SYBR Green Jump Start Taq ready mix (Sigma-Aldrich, Oakville, ON, Canada), as previously described [23, 24]. Primer forward and reverse sequences for genes of interest as well as annealing temperatures are shown in **Table S2**. mRNA levels were calculated using the standard curve method (Rotor Gene 3000) and normalized to GAPDH as a housekeeping gene. Genes selected represented important processes expected to be involved in the reduced body weight and fat mass as well as in the conversion of white to brown AT in response to physical exercise alone or associated with citrulline. All measurements were performed in duplicate, and then averaged [23, 24].

**References**

1. Christopher A, Kraft E, Olenick H, Kiesling R, Doty A. The reliability and validity of the Timed Up and Go as a clinical tool in individuals with and without disabilities across a lifespan: a systematic review. Disabil Rehabil. 2021;43:1799-813.

2. Podsiadlo D, Richardson S. The timed "Up & Go": a test of basic functional mobility for frail elderly persons. Journal of the American Geriatrics Society. 1991;39:142-8.

3. Mathias S, Nayak US, Isaacs B. Balance in elderly patients: the "get-up and go" test. Archives of physical medicine and rehabilitation. 1986;67:387-9.

4. Choi YM, Dobson F, Martin J, Bennell KL, Hinman RS. Interrater and intrarater reliability of common clinical standing balance tests for people with hip osteoarthritis. Phys Ther. 2014;94:696-704.

5. Springer BA, Marin R, Cyhan T, Roberts H, Gill NW. Normative values for the unipedal stance test with eyes open and closed. J Geriatr Phys Ther. 2007;30:8-15.

6. Yanagawa N, Shimomitsu T, Kawanishi M, Fukunaga T, Kanehisa H. Relationship between performances of 10-time-repeated sit-to-stand and maximal walking tests in non-disabled older women. Journal of physiological anthropology. 2016;36:2.

7. Csuka M, McCarty DJ. Simple method for measurement of lower extremity muscle strength. Am J Med. 1985;78:77-81.

8. Chung MM, Chan RW, Fung YK, Fong SS, Lam SS, Lai CW, et al. Reliability and validity of Alternate Step Test times in subjects with chronic stroke. Journal of rehabilitation medicine. 2014;46:969-74.

9. Berg KO, Wood-Dauphinee SL, Williams JI, Maki B. Measuring balance in the elderly: validation of an instrument. Canadian journal of public health = Revue canadienne de sante publique. 1992;83 Suppl 2:S7-11.

10. Rikli RE, Jones CJ. The Reliability and Validity of a 6-Minute Walk Test as a Measure of Physical Endurance in Older Adults. Journal of Aging and Physical Activity. 1998;6:363-75.

11. ATS statement: guidelines for the six-minute walk test. American journal of respiratory and critical care medicine. 2002;166:111-7.

12. Aubertin-Leheudre M, Audet M, Goulet ED, Dionne IJ. HRT provides no additional beneficial effect on sarcopenia in physically active postmenopausal women: a cross-sectional, observational study. Maturitas. 2005;51:140-5.

13. Guralnik JM, Ferrucci L, Pieper CF, Leveille SG, Markides KS, Ostir GV, et al. Lower extremity function and subsequent disability: consistency across studies, predictive models, and value of gait speed alone compared with the short physical performance battery. J Gerontol A Biol Sci Med Sci. 2000;55:M221-31.

14. Ostir GV, Volpato S, Fried LP, Chaves P, Guralnik JM. Reliability and sensitivity to change assessed for a summary measure of lower body function: results from the Women's Health and Aging Study. J Clin Epidemiol. 2002;55:916-21.

15. St-Jean-Pelletier F, Pion CH, Leduc-Gaudet JP, Sgarioto N, Zovile I, Barbat-Artigas S, et al. The impact of ageing, physical activity, and pre-frailty on skeletal muscle phenotype, mitochondrial content, and intramyocellular lipids in men. Journal of cachexia, sarcopenia and muscle. 2017;8:213-28.

16. Skelton DA, Kennedy J, Rutherford OM. Explosive power and asymmetry in leg muscle function in frequent fallers and non-fallers aged over 65. Age and ageing. 2002;31:119-25.

17. Bassey EJ, Short AH. A new method for measuring power output in a single leg extension: feasibility, reliability and validity. European Journal of Applied Physiology and Occupational Physiology. 1990;60:385-90.

18. Luhrmann PM, Herbert BM, Gaster C, Neuhauser-Berthold M. Validation of a self-administered 3-day estimated dietary record for use in the elderly. European journal of nutrition. 1999;38:235-40.

19. Colbert LH, Matthews CE, Havighurst TC, Kim K, Schoeller DA. Comparative validity of physical activity measures in older adults. Medicine and science in sports and exercise. 2011;43:867-76.

20. Dulac M, Leduc-Gaudet JP, Reynaud O, Ayoub MB, Guerin A, Finkelchtein M, et al. Drp1 knockdown induces severe muscle atrophy and remodelling, mitochondrial dysfunction, autophagy impairment and denervation. The Journal of physiology. 2020;598:3691-710.

21. Dulac M, Leduc-Gaudet JP, Cefis M, Ayoub MB, Reynaud O, Shams A, et al. Regulation of muscle and mitochondrial health by the mitochondrial fission protein Drp1 in aged mice. The Journal of physiology. 2021;599:4045-63.

22. Gouspillou G, Sgarioto N, Kapchinsky S, Purves-Smith F, Norris B, Pion CH, et al. Increased sensitivity to mitochondrial permeability transition and myonuclear translocation of endonuclease G in atrophied muscle of physically active older humans. FASEB journal : official publication of the Federation of American Societies for Experimental Biology. 2014;28:1621-33.

23. Mauriege P, Joanisse DR, CasparBauguil S, Cartier A, Lemieux I, Bergeron J, et al. Gene expression of different adipose tissues of severely obese women with or without a dysmetabolic profile. Journal of physiology and biochemistry. 2015;71:719-32.

24. Mauriege P, Karelis AD, Taleb N, Clement AA, Joanisse DR. Comparing an adiposopathy approach with four popular classifications schemes to categorize the metabolic profile of postmenopausal women. Journal of physiology and biochemistry. 2020;76:609-22.
