# Supplementary material for: Impact of high‐intensity interval training with or without l‐citrulline on physical performance, skeletal muscle, and adipose tissue in obese older adults
Source: J Cachexia Sarcopenia Muscle. 2022 Mar 7;13(3):1526–40. doi: 10.1002/jcsm.12955 (PMC9178162; doi:10.1002/jcsm.12955)
Supplement: Supplementary file 2 — Figure S1: Study Overview Figure S2: Impact of HIIT with or without CIT on functional capacities Figure S3: Impact of HIIT with or without CIT on body composition Figure S4: Impact of HIIT with or without CIT on muscle strength and power and quality Figure S5: Impact of HIIT with or without CIT on TFAM and TOM20 content Figure S6: OXPHOS subunit content in HIIT‐PLA and HIIT‐CIT at baseline (pre‐intervention) Figure S7: Impact of HIIT with or without CIT on markers of mitochondrial dynamics and mitophagy [file JCSM-13-1526-s002.pdf]

Figure S1: Study Overview

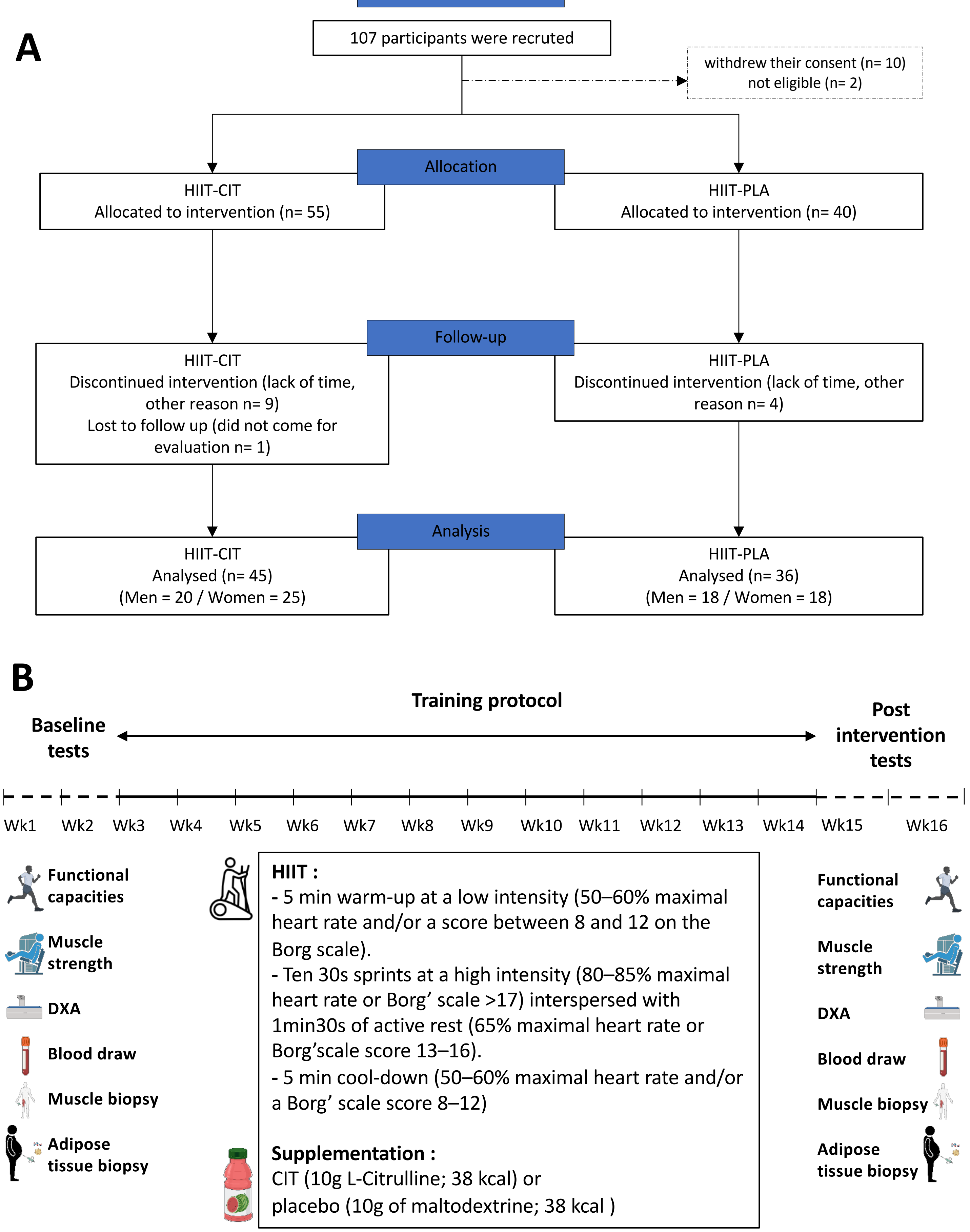

(A) Diagram of the study, (B) overview of the intervention. Parts of this figure were created with BioRender.com.

Figure S2: Impact of HIIT with or without CIT on functional capacities

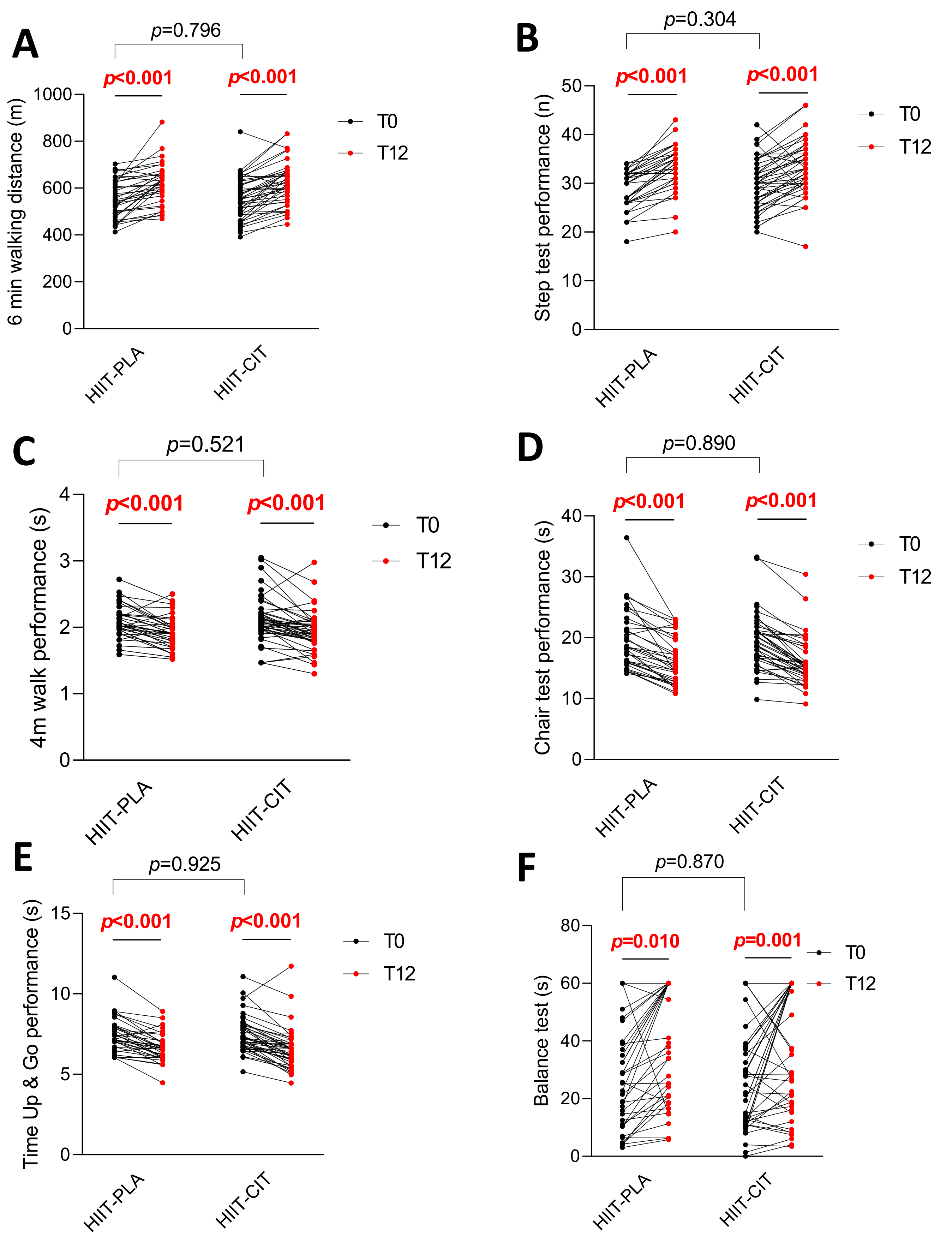

The impact of 12 weeks of HIIT without (HIIT-PLA) or with CIT (HIIT-CIT) on the performance at the 6 min walking test (A), step test (B), 4m walk test (C), chair test (D), Time Up and Go test (E) and balance test (F). Differences between HIIT-PLA and HIIT-CIT at baseline (T0) were tested with an unpaired bilateral student t-test. Differences from T0 to T12 (i.e. pre vs post intervention) were tested by a Sidak post hoc test that followed 2-way repeated measures ANOVAs. All significant p-values ($p<0.05$) are highlighted in bold and in red.

Figure S3: Impact of HIIT with or without CIT on body composition

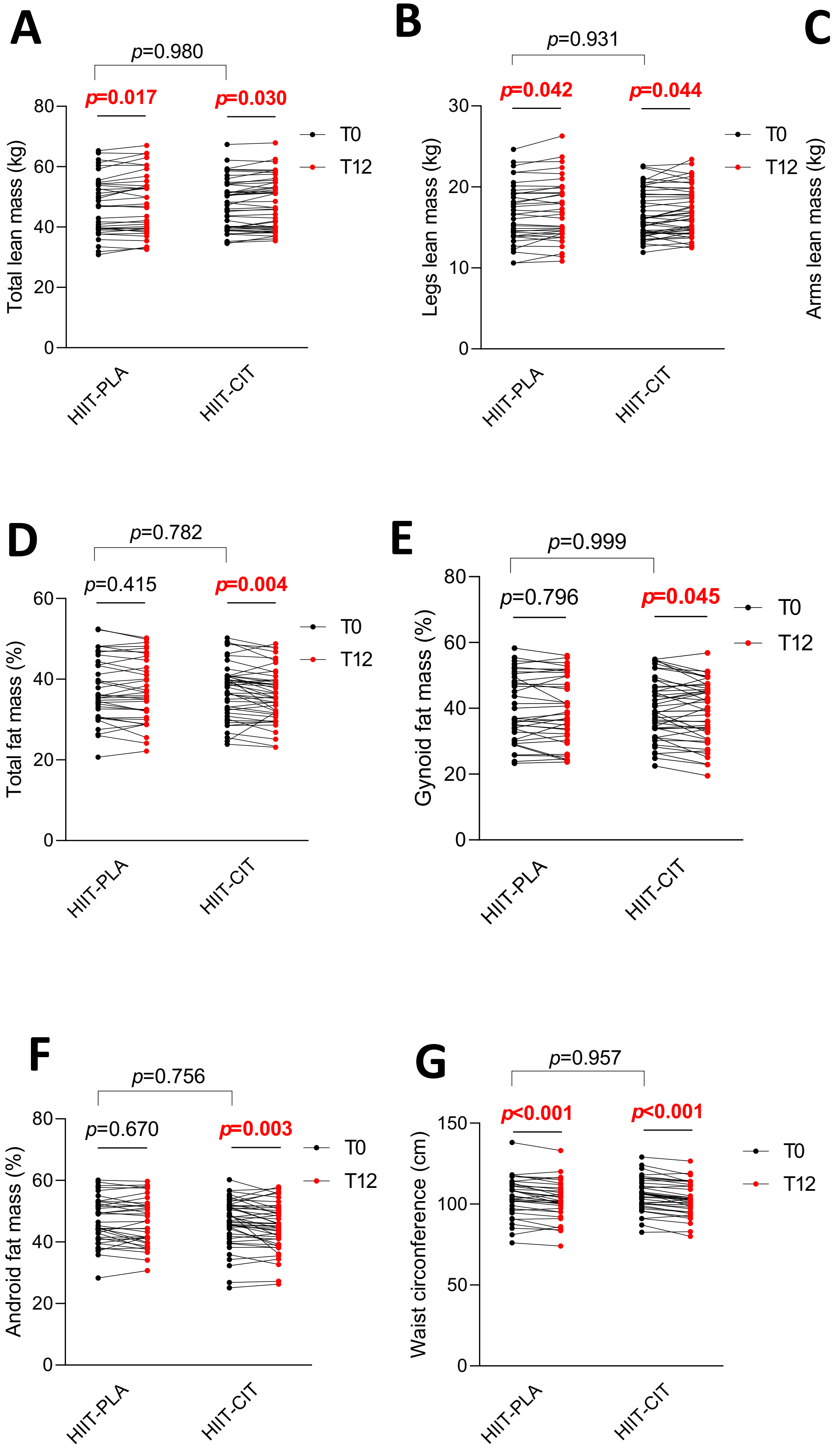

The impact of 12 weeks of HIIT without (HIIT-PLA) or with CIT (HIIT-CIT) on total lean mass (A), leg lean mass (B), arm lean mass (C), total fat mass (D), gynoid fat mass (E), android fat mass (F) and waist circumference (G). Differences between HIIT-PLA and HIIT-CIT at baseline (T0) were tested with an unpaired bilateral student t-test. Differences from T0 to T12 (i.e. pre vs post intervention) were tested by a Sidak post hoc test that followed 2-way repeated measures ANOVAs All significant p-values ($p<0.05$) are highlighted in bold and in red.

Figure S4: Impact of HIIT with or without CIT on muscle strength and power and quality

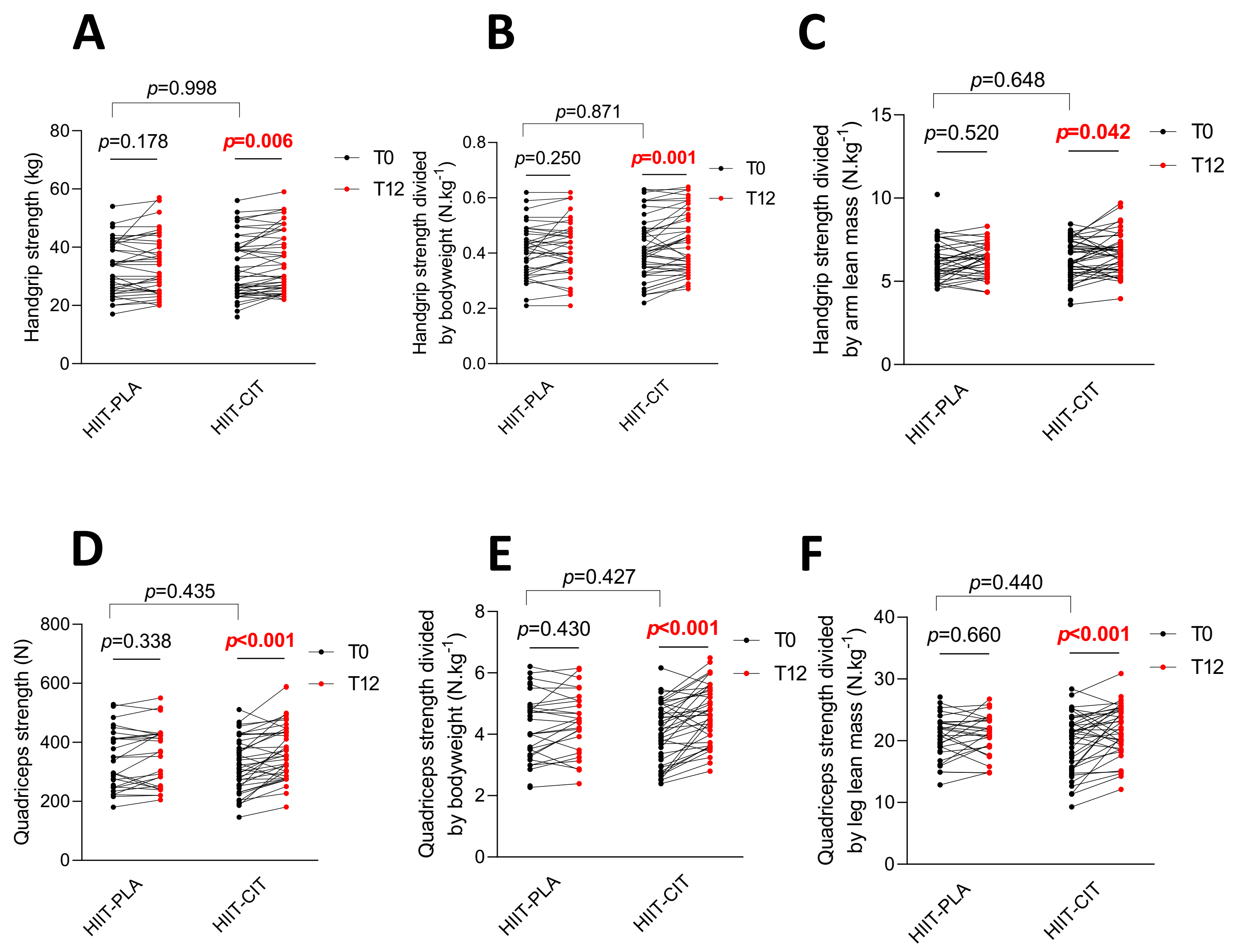

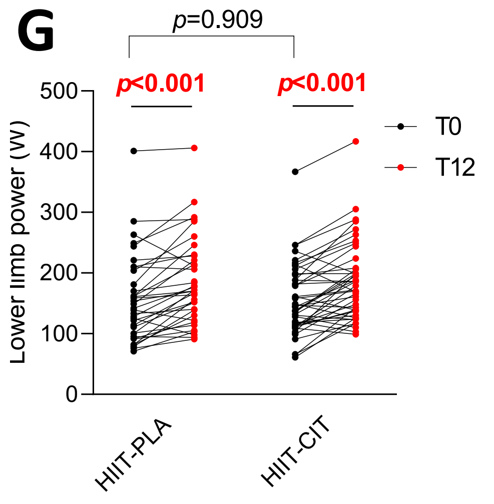

The impact of 12 weeks of HIIT without (HIIT-PLA) or with CIT (HIIT-CIT) on handgrip strength (A), handgrip strength normilized to body weight (B), handgrip strength normilized to arm lean mass (C), quadriceps strength (D), quadriceps strength normilized to body weight (E), quadriceps strength normilized to led lean mass (F) and lower limb power (G). Differences between HIIT-PLA and HIIT-CIT at baseline (T0) were tested with an unpaired bilateral student t-test. Differences from T0 to T12 (i.e. pre vs post intervention) were tested by a Sidak post hoc test that followed 2-way repeated measures ANOVAs All significant p-values (p<0.05) are highlighted in bold and in red.

Figure S5 : Impact of HIIT with or without CIT on TFAM andTOM20 content

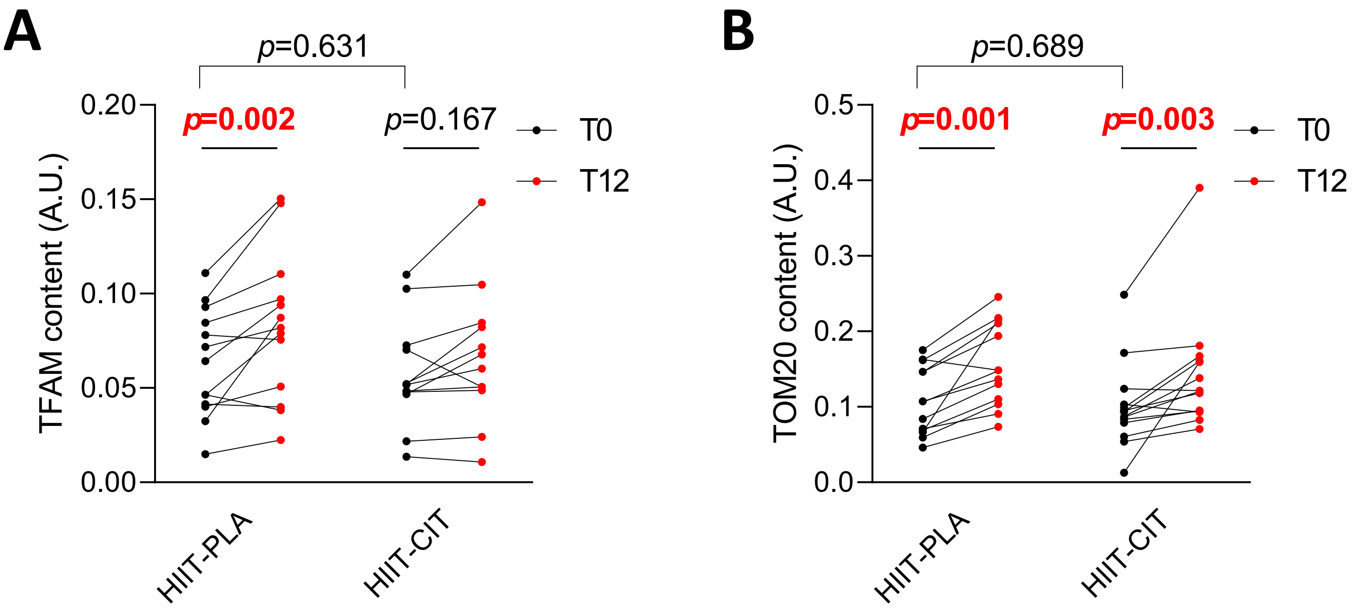

Fixed effects (type III)	p-value
Supplementation	0.419
Time	**<0.001**
Supplementation x Time	0.1802

Fixed effects (type III)	p-value
Supplementation	0.629
Time	**< 0.001**
Supplementation x Time	0.748

The impact of 12 weeks of HIIT without (HIIT-PLA) or with CIT (HIIT-CIT) on TFAM content (A) and TOM20 content (B). Differences between HIIT-PLA and HIIT-CIT at baseline (T0) were tested with an unpaired bilateral student t-test. Tables under each graph presents the results of the 2-way repeated measures ANOVAs. Differences from T0 to T12 (i.e. pre vs post intervention) were tested by a Sidak post hoc test. All significant p-values ($p<0.05$) are highlighted in bold and in red.

Figure S6 : OXPHOS subunit content in HIIT-PLA and HIIT-CIT at baseline (pre-intervention)

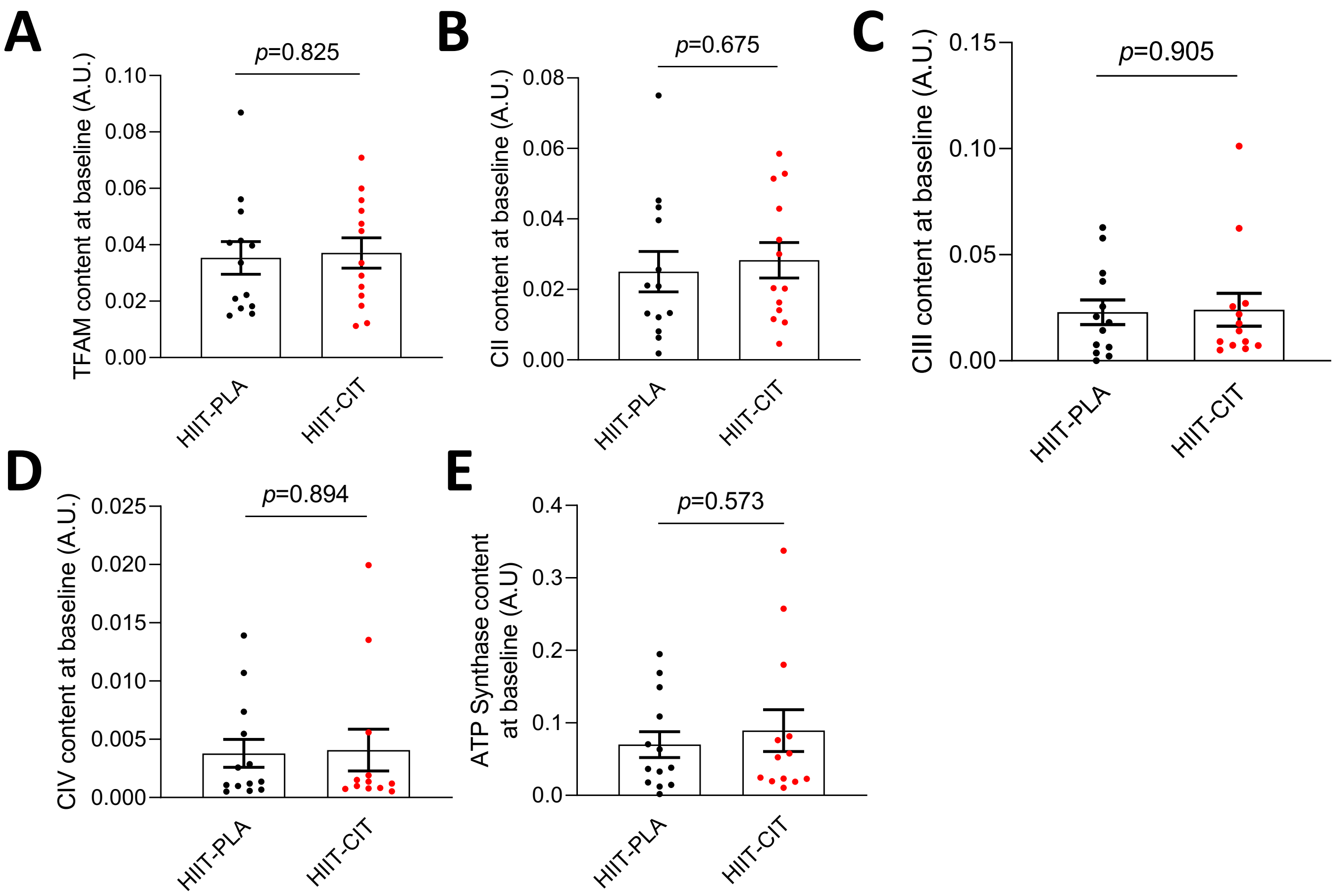

The content of representative subunits of Complex I (CI; A), Complex II (CII; B) , Complex III (CIII; C), Complex IV (CIV; D) and ATP synthase (ATPs; E) between HIIT-PLA and HIIT-CIT at baseline (T0; pre-intervention). Difference between HIIT-PLA and HIIT-CIT at baseline were tested with unpaired bilateral student t-tests.

Figure S7: Impact of HIIT with or without CIT on markers of mitochondrial dynamics and mitophagy

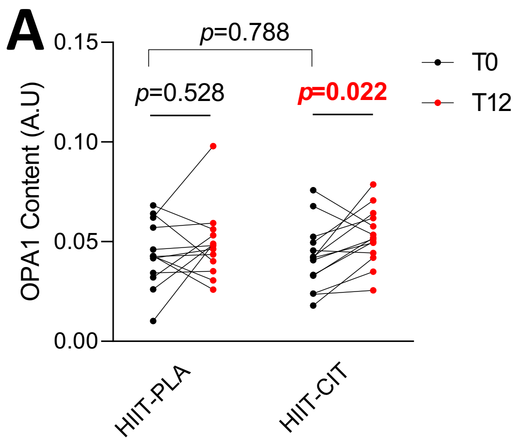

Fixed effects (type III)	p-value
Supplementation	0.841
Time	**0.030**
Supplementation x Time	0.417

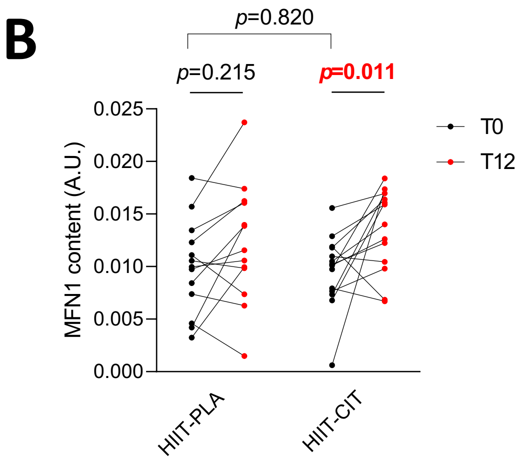

Fixed effects (type III)	p-value
Supplementation	0.704
Time	**0,003**
Supplementation x Time	0.335

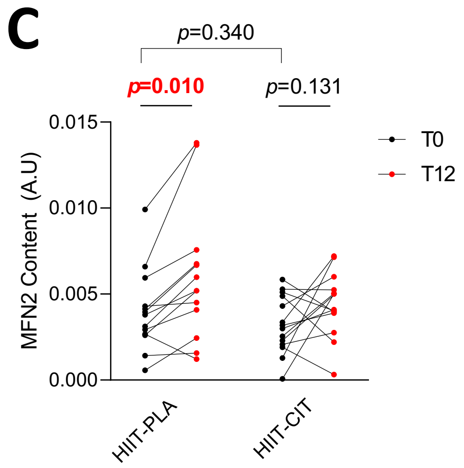

Fixed effects (type III)	p-value
Supplementation	0.127
Time	**<0.001**
Supplementation x Time	0.459

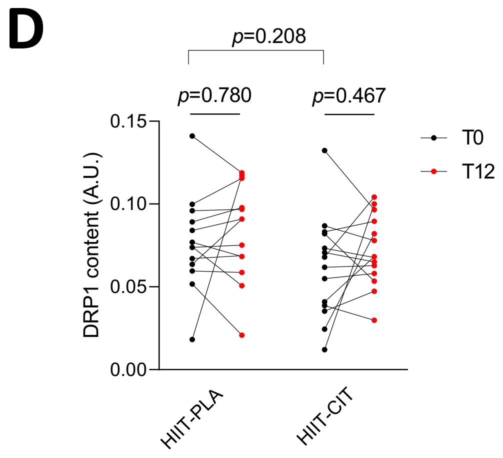

Fixed effects (type III)	p-value
Supplementation	0.149
Time	0.226
Supplementation x Time	0.748

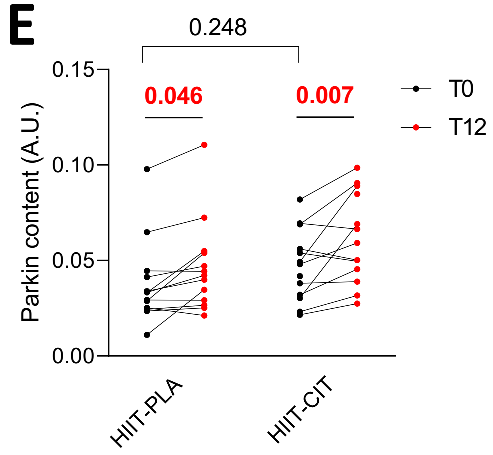

Fixed effects (type III)	p-value
Supplementation	0.190
Time	**0.001**
Supplementation x Time	0.351

The impact of 12 weeks of HIIT without (HIIT-PLA) or with CIT (HIIT-CIT) on Opa1 content (A), Mfn1 content (B), Mfn2 content (C), Drp12 content (D) and Parkin content (E). Differences between HIIT-PLA and HIIT-CIT at baseline (T0) were tested with an unpaired bilateral student t-test. Tables under each graph presents the results of the 2-way repeated measures ANOVAs. Differences from T0 to T12 (i.e. pre vs post intervention) were tested by a Sidak post hoc test. All significant p-values ($p<0.05$) are highlighted in bold and in red.
