# Supplementary material for: Impact of high‐intensity interval training with or without l‐citrulline on physical performance, skeletal muscle, and adipose tissue in obese older adults
Source: J Cachexia Sarcopenia Muscle. 2022 Mar 7;13(3):1526–40. doi: 10.1002/jcsm.12955 (PMC9178162; doi:10.1002/jcsm.12955)
Supplement: Supplementary file 3 — Table S1: List of antibodies Table S2: Primer sequences and qPCR conditions for genes of interest. Table S3: Impact of HIIT with and without CIT on blood parameters [file JCSM-13-1526-s001.docx]

**Table S1:** List of antibodies

| **Antibody** | **Source / Product no.** | **Dilution** |
| --- | --- | --- |
| Rabbit anti-total Drp1 | Abcam #ab184247 | 1/1000 |
| Rabbit anti-MFN1 | Abcam #ab13798 | 0,5ug/ml |
| Rabbit anti-MFN2 | Abcam #ab124773 | 1/1000 |
| Rabbit anti-OPA1 | Abcam #ab42364 | 1ug/ml |
| Mouse anti-OXPHOS | Abcam #ab110413 | 1/500 |
| Rabbit anti-TOM20 | Abcam #ab186735 | 1/5000 |
| Rabbit anti-TFAM | Abcam #ab131607 | 1/1000 |
| Mouse anti-Parkin | Abcam #ab77924 | 1/1000 |
| Goat anti-mouse IgG | Abcam #ab6728 | 1/5000 |
| Goat anti-rabbit IgG | Abcam #ab6721 | 1/5000 |

**Table S2: Primer sequences and qPCR conditions for genes of interest.**

| Gene | Annealing T | Primers |
| --- | --- | --- |
| PPAR-γ2 | 60°C | F5’-GCGATTCCTTCACTGATAC-3’  R5’-CTTCCATTACGGAGAGATCC-3’ |
| HSL | 64^°^C | F5’-CCCCCATCATCTCCATCGACTA-3’  R5’-GCTGACACACTTGGAGAGCACA-3’ |
| ATGL | 61^°^C | F5’-AATGTCTGCAGCGGTTTCATCC-3’  R5’-TGCACATCTCTCGCAGCACCAG-3’ |
| Cgi-58/ABHD5 | 58^°^C | F5’-GGGAGAGGCTCCCCTCAG-3’  R5’-GCCAACCAGTTAGCCATCCT-3’ |
| PLIN-1 | 57^°^C | F5’-TGGAGACTGAGGAGAACAAG-3’  R5’-ATGTCACAGCCGAGATGG-3’ |
| PLIN-3 | 55^°^C | F5’-TTGTGTCGTCTAAGGTGTCG-3’  R5’-GCCCAGACGTACGAAGTAGC-3’ |
| LPL | 64^°^C | F5-ACACAGCTGAGGACACTTGC-3’  R5-CACTGGGTAATGCTCCTGAG-3’ |
| FABP-4 | 60^°^C | F5’-GCATGGCCAAACCTAACATGA-3’  R5’-CCTGGCCCAGTATGAAGGAAA-3’ |
| FAT/CD36 | 55^°^C | F5’-TGGATATTGAACCTATAACTGG-3’  R5’-AACATCACCACACCAACAC-3’ |
| DGAT-1 | 58^°^C | F5’-TCATCTGGCTCATCTTCTTCTAC-3’  R5’-AGGCCAGTGGGATCTGAG-3’ |
| DGAT-2 | 57^°^C | F5'-ATAAAGTGTTGCGCTCCGGG-3'  R5'-AGTAGGCGGCTATGAGGGTC-3' |
| CIDEA | 55^°^C | F5-CTCATCAGGCCCCTGACATT-3’  R5’ CTATTCCCGACCTCTTCGGC-3’ |
| PGC-1α | 52^°^C | F5’-GGGATGATGGAGACAGCTATG-3’  R5’-ATACTTGCTCTTGGTGGAAGC-3’ |
| CPT-1β | 55^°^C | F5’-CTCTTCCAGAAGGCTGCTAAG-3’  R5’-CATCTGCTACAGGGCCAAAG-3’ |
| PRDM16 | 55^°^C OK | F5’-CAGCACGGTGAAGCCTTTC-3’  R5’-GCGTGCATCCGCTTGTG-3’ |
| UCP-1 | 56^°^C | F5’-CTCTCAGGATCGGCCTCT-3’  R5-GAGTAGTCCCTTTCCAAAGACC-3’ |
| GAPDH | 64^°^C | F5’-ATCACCCCTTCATTGACCTCAAC-3’  R5’-GGTTCACACCCATGACGAACATG-3’ |

qPCR: quantitative polymerase chain reaction; F: Forward; R: Reverse; T: Temperature; PPAR-γ2: peroxisome proliferator-activated receptor γ2; HSL: Hormone Sensitive Lipase; ATGL: Adipose TriGlyceride Lipase; Cgi-58/: Comparative Gene Identification-58/α/β-hydrolase domain containing 5; PLIN-1/-3: perilipin-1/-3; FABP-4: Fatty Acid Binding Protein-4; LPL: LipoProtein Lipase; DGAT-1/-2: DiacylGlycerol Acyl Transferase-1/-2; FAT/CD36: Fatty Acid Transferase/Cluster of Differentiation 36; CIDEA: cell death-inducing DFFA-like effector a; PGC-1α: Peroxisome proliferator-activated receptor-gamma coactivator-1alpha; CPT-1β: Carnityl PalmitolylTransferase-1β; PRDM16: PR domain containing 16; UCP-1: uncoupling protein-1; GAPDH: GlycerAldehyde Phosphate Dehydrogenase.

**Table S3:** Impact of HIIT with and without CIT on blood parameters

| **Variable** | **HIIT-PLA**  **T0** | **HIIT-PLA**  **T12** | **HIIT-CIT**  **T0** | **HIIT-CIT**  **T12** | **Group effect** | **Time effect** | **Time * group effect** |
| --- | --- | --- | --- | --- | --- | --- | --- |
| **Glucose (mmol. L^-1^)** | 5.9 ± 1.3 | 6.0 ± 1.5 | 5.5 ± 0.7**^d^** | 5.7 ± 0.7 **^c^** | 0.133 | **0.030** | 0.316 |
| **Insulin (pmol. L^-1^)** | 47.4 ± 23.8 | 49.5 ± 29.3 | 51.3 ± 28.3 | 51.9 ± 27.0 | 0.543 | 0.480 | 0.724 |
| **HOMA (A.U.)** | 2.1 ± 1.8 | 2.2 ± 1.4 | 2.2 ± 1.3 | 2.3 ± 1.4 | 0.813 | 0.242 | 0.906 |
| **Quicki (A.U.)** | 0.35 ± 0.04 | 0.35 ± 0.04 | 0.35 ± 0.04 | 0.35 ± 0.03 | 0.700 | 0.253 | 0.966 |
| **Total IGF1 (ng.mL^-1^)** | 87.3 ± 22.3 | 89.6 ± 22.8 | 92.9 ± 28.2 | 95.7 ± 31.2 | 0.428 | 0.218 | 0.979 |
| **IGFBP3 (μg.mL^-1^)** | 1.8 ± 0.3 | 1.9 ± 0.3 | 2.0 ± 0.5 | 2.0 ± 0.5 | 0.289 | 0.275 | 0.271 |
| **IGF1 / IGFBP3 (molar ratio % )** | 0.18 ± 0.04 | 0.17 ± 0.04 | 0.17 ± 0.04 | 0.18 ± 0.04 | 0.651 | 0.322 | 0.061 |
| **Cholesterol total (mmol.L^-1^)** | 5.2 ± 1.3 | 5.2 ± 1.2 | 5.4 ± 1.1 | 5.1 ± 1.0 | 0.827 | 0.123 | 0.168 |
| **HDL (mmol. L^-1^)** | 1.5 ± 0.4 | 1.5 ± 0.4 | 1.5 ± 0.3 | 1.5 ± 0.3 | 0.954 | 0.881 | 0.190 |
| **LDL (mmol. L^-1^)** | 3.1 ± 1.1 | 3.1 ± 1.0 | 3.2 ± 1.0 | 3.1 ± 0.8 | 0.691 | 0.439 | 0.114 |
| **Non-HDL (mmol. L^-1^)** | 3.8 ± 1.2 | 3.7 ± 1.1 | 3.9 ± 1.0 | 3.7 ± 1.0 | 0.782 | 0.119 | 0.219 |
| **Triglycerides (mmol. L^-1^)** | 1.6 ± 0.7 **^b^** | 1.4 ± 0.6 **^a^** | 1.4 ± 0.8 | 1.4 ± 0.7 | 0.756 | **0.008** | 0.260 |
| **Free fatty acids (mmol. L^-1^)** | 0.50 ± 0.15 | 0.49 ± 0.23 | 0.45 ± 0.18 | 0.44 ± 0.15 | 0.160 | 0.727 | 0.868 |
| **Adiponectin (μg.mL^-1^)** | 14.2 ± 7.3 | 13.8 ± 7.4 | 13.7 ± 8.4 | 14.0 ± 10.1 | 0.897 | 0.962 | 0.456 |
| **Leptin (ng.mL^-1^)** | 23.4 ± 19.4 | 25.9 ± 19.8 | 27.5 ± 19.5 | 23.0 ± 17.8 | 0.608 | 0.653 | **0.053** |
| Total adiponectin/leptin ratio (μg/ng) | 1.19 ± 1.48 | 0.94 ± 0.94 | 0.82 ± 0.72 | 1.03 ± 0.91 | 0.207 | 0.655 | **0.015** |
| **Ferritin (mmol. L^-1^)** | 140.2 ± 117.9**^c^** | 124.3 ± 99.32**^d^** | 86.0 ± 54.5**^a^****^d^** | 67.3 ± 46.6**^bc^** | **0.012** | **0.001** | 0.689 |

a: p<0.05 vs HIIT-PLA T0

b: p<0.05 vs HIIT-PLA T12

c: p<0.05 vs HIIT-CIT T0

d: p<0.05 vs HIIT-CIT T12

Data are mean ± SD.
